# Two-dimensional Cd(ii) coordination polymer encapsulated by Tb^3+^ as a reversible luminescent probe for Fe^3+^†

**DOI:** 10.1039/c9ra06639j

**Published:** 2019-10-29

**Authors:** Yuandi Wu, Meihua Lin, Dongyang Liu, Ming Liu, Jing Qian

**Affiliations:** College of Chemistry, Tianjin Normal University Tianjin 300387 P. R. China qianjinger@aliyun.com; Tianjin Key Laboratory of Structure and Performance for Functional Molecules, Tianjin Normal University Tianjin 300387 P. R. China; Key Laboratory of Inorganic–Organic Hybrid Functional Materials Chemistry, Tianjin Normal University, Ministry of Education Tianjin 300387 P. R. China

## Abstract

A two-dimensional luminescent cadmium(ii) coordination polymer, [Cd(modbc)_2_]_*n*_ (Cd-P); modbc = 2-methyl-6-oxygen-1,6-dihydro-3,4′-bipyridine-5-carbonitrile, was successfully synthesized by a solvothermal reaction and fully characterized. Cd-P exhibited excellent luminescence emission, and detected Cu^2+^, Co^2+^, Fe^2+^, Hg^2+^, Ni^2+^ and Fe^3+^ ions with high sensitivity and showed good anti-interference performance. After encapsulation of Tb^3+^ ions in Cd-P, the as-obtained fluorescent functionalized Tb^3+^@Cd-P maintained distinct chemical stabilities in different pHs and metal salt solutions. Subsequently, we explored the potential application of Tb^3+^@Cd-P as a probe for Fe^3+^ ions. A new and convenient method for individual identification of Fe^3+^ ions by the combination of Cd-P and Tb^3+^@Cd-P was successfully established. A possible sensing mechanism is discussed in detail.

## Abstract 2

A new and convenient method for individual identification of Fe^3+^ ions by the combination of Cd-P and Tb^3+^@Cd-P was successfully established.

## Graphical abstract

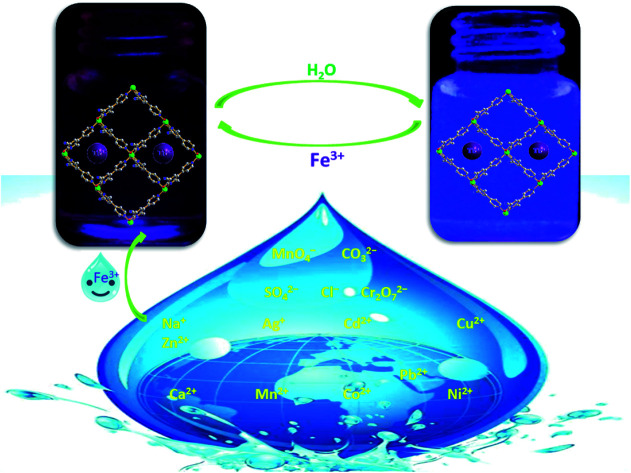

## 1. Introduction

Chemists have been highly successful at developing detection methods for anions, cations, small organic molecules and biological macromolecules. Fluorescent organogels have become novel and promising materials, especially in sensor applications.^1,2^ Metal–organic frameworks (MOFs) can be defined as one of the ideal candidates for chemical sensors.^3^ Luminescent coordination polymers (CPs) as chemosensors, have also attracted more and more attention for the selective and sensitive detection of some explosives,^4,5^ organic compounds,^6,7^ inorganic ions,^8,9^*etc.*, through “turn on”^10^ or “turn off”^11^ sensing. Among them, rapid selection and sensitive detection of Fe^3+^ ions has aroused widespread interest because Fe^3+^ ions have important cell functions such as hemoglobin formation and play a vital role in biological systems.^12–14^ Its deficiency or excess over the normal allowable limit can lead to physical diseases such as diabetes, anemia, arthritis, mental decline, cancer and so on.^15,16^ In addition, with the rapid development of industry, a large number of harmful inorganic ions is being released into the environment, and causing adverse effects on people's health.^17–19^ In a word, not only is Fe^3+^ recognized as an industrial pollutant but also it plays a significant role in living organisms. Therefore, it is an urgent problem to explore the high effective probes to detect Fe^3+^.^20–23^

3D microporous CPs, which can produce significant fluorescence signals and visible emission colors, and have become the most reported chemical sensors.^4,7–11,24^ At present, compared with the transition-metal-based CPs, Ln-CPs have aroused great interest due to their unique optical characteristics such as large stokes shift, high color purity, and long fluorescence lifetime *via* the “antenna effect” obtained from the 4f–4f electron transitions.^7,8,25,26^ Recently, an alternative strategy for constructing Ln-CPs and optimizing photoluminescence was proposed by doping lanthanide ions to CPs *via* post-synthesis method (PSM).^27–29^ Any desired fluorescent probes can be acquired by modifying the molar ratio of the starting reactants.^30–33^ In the construction of CPs for the above applications, poly-carboxylic acid and N-donor ligands have been widely chosen as building blocks. However, the achievement of a fast response, practicability, and reproducible performance for Fe^3+^ detection using fluorescence is still challenging.^34^

**Scheme 1:**
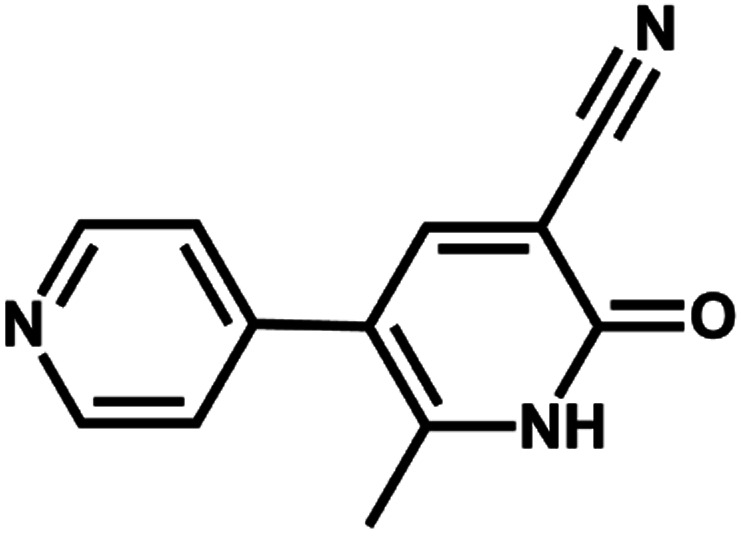
The structure of modbc.

In fact, it was rarely reported that low-dimensional chemical sensors with one-dimensional or two-dimensional structures were considered for selective detection of inorganic ions.^35,36^ In this study, asymmetric unflexible 2-methyl-6-oxygen-1,6-dihydro-3,4′-bipyridine-5-carbonitrile (modbc) containing coordination N and O atoms, was utilized as an anionic ligand (Scheme 1). Then, we report a multifunctional highly luminescent [Cd(modbc)_2_]_*n*_ (Cd-P), and obtain a Ln-decorated CP Tb^3+^@Cd-P. Cd-P was fully characterized by IR spectroscopy, elemental analysis, single crystal, XPS, powder X-ray diffraction, thermal and photoluminescence properties. Moreover, selective and sensing properties of Cd-P and Tb^3+^@Cd-P were investigated in H_2_O for Fe^3+^ ions in detail.

## 2. Experimental section

### 2.1. Synthesis of [Cd(modbc)_2_]_*n*_ (Cd-P)

Cd-P was obtained by one-pot solvothermal synthesis from CdSO_4_ (0.05 mmol, 0.008 g), modbc (0.1 mmol, 0.021 g) in 10 mL of water/DMF (4 : 1). The reactants were placed in a 25 ml reactor, and heated to 120 °C for 72 h under self-generated pressure, then cooled to atmospheric temperature at a rate of 2.0 °C h^−1^. Light yellow rodlike-shaped crystals suitable for X-ray analysis were obtained, which were filtered, washed and dried (Tables S1 and S2†). Total yield of Cd-P was *ca.* 54% based on CdSO_4_. Anal. Calcd for Cd_0.50_C_12_H_8_N_3_O: C, 54.1; H, 3.03; N, 15.77%. Found: C, 53.59; H, 3.47; N, 15.34%. FT-IR (KBr, cm^−1^): 3377.05 (*vs.*), 2982.1 (s), 2215.19 (m), 1601.95 (s), 1386.11 (s), 1109.61 (*vs.*), 983.56 (*vs.*), 856.65 (s), 617.75 (m), 541.33 (*vs.*) (Fig. S1†).

### 2.2. Preparation of Tb^3+^@Cd-P

Tb^3+^@Cd-P was prepared by adding 100 mg powder of Cd-P to the aqueous solution of Tb(NO_3_)_3_ soaking for 24 h, and separated by centrifugation and washed with deionized water to remove the remaining Tb^3+^. The collected crystal powder was dried for 12 h under vacuum conditions of 60 °C.

### 2.3. Experiment of luminescent detection

Cd-P and Tb^3+^@Cd-P (0.01 mM) were well-dispersed in 5 mM Tris–HCl/NaCl buffer (pH 7.0) by sonicating for 0.5 h to obtain a solution, which were used for luminescent experiments. The aqueous solutions of nitrate salts or chloride of Na^+^, Ag^+^, Cd^2+^, Cu^2+^, Zn^2+^, Pb^2+^, Ca^2+^, Mn^2+^, Co^2+^, Ni^2+^, Hg^2+^, Fe^3+^ (1.0 × 10^−3^ M) were prepared for luminescent experiments. Generally, ferrous salt is easy to be oxidized in the air, but it is relatively stable and not easy to be oxidized after forming double salt, so we chose double salt (NH_4_)_2_Fe(SO_4_)_2_ to prepare the aqueous solution of Fe^2+^. In addition, the same concentrations (1.0 × 10^−3^ M) of aqueous solutions containing potassium salts of MnO_4_^−^, CO_3_^2−^, SO_4_^2−^, Cl^−^, and Cr_2_O_7_^2−^ were also prepared. The Stern–Volmer equation: *I*_0_/*I* = 1 + *K*_sv_[Q] was applied to judge the quenching effect.^37^ The detection limit was calculated according to 3*σ*/*k* recommended by IUPAC, where the standard deviation, *σ* value was estimated by fifteen repeated fluorescent measurements of Cd-P and Tb^3+^@Cd-P, and *k* value was obtained using a calibration curve of *I vs.* [Q].^38^ The fluorescence stability of Cd-P and Tb^3+^@Cd-P in the aqueous solution at different pH values were investigated.

## 3. Results and discussion

### 3.1. Structural description

**Fig. 1:**
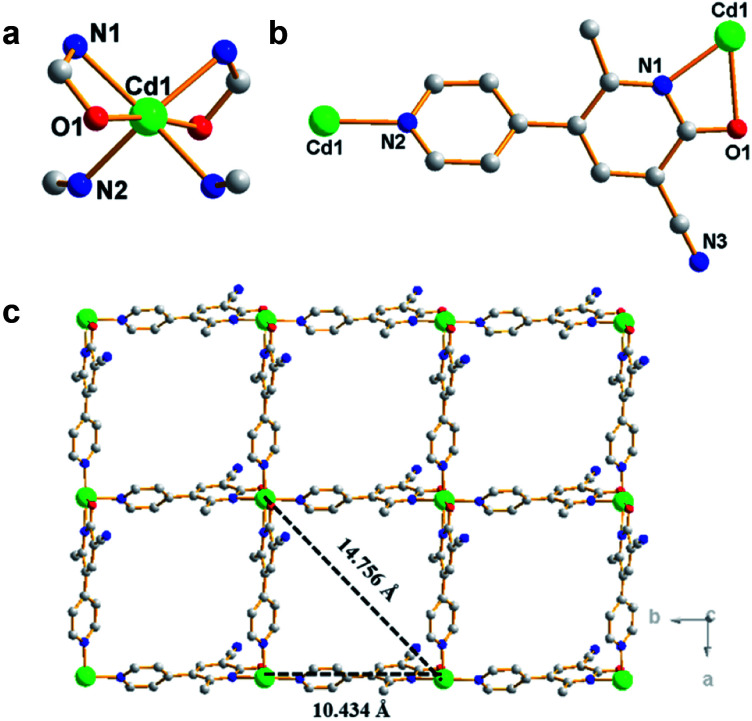
(a) Coordination environment of Cd^2+^ ion in Cd-P. (b) Coordination mode of modbc molecule. (c) Two-dimensional reticulated structure of Cd-P along the *c* axis; red, O; gray, C; green, Cd1; blue, N. All H atoms are omitted for clarity.

By one-pot solvothermal method, CdSO_4_ combined with modbc produced a 2D framework at about 54% yield. Single-crystal X-ray diffraction shows that Cd-P crystallizes in the *P̄*4_3_2_1_2 space group. The symmetric unit consists of one Cd^2+^ ion and two modbc anions. A six-coordinated Cd^2+^ ion exhibits a distorted octahedron geometry, which is completed by two oxygen atoms and four nitrogen atoms from different modbc molecules (Fig. 1a). Each modbc molecule adopts tridentate mode (Fig. 1b). The Cd–N and Cd–O bond lengths fall in the reasonable range of 2.269–2.501 Å, and adjacent Cd^2+^ ions are bridged by modbc ligands into the cadmium chain. The structural feature of Cd-P is that each cadmium chain serves as a secondary building unit and is further connected by a modbc– ligand into a 2D framework, containing tetranuclear homometallic Cd_4_(modbc)_4_ cycles, with different Cd1⋯Cd1 distances of 10.434 and 14.756 Å (Fig. 1c).

### 3.2. Property characterization

As shown in Fig. S2,† thermogravimetric analysis (TGA) of Cd-P was measured to evaluate its thermal stability at ≤450 °C. Further, powder X-ray diffraction (PXRD) of Cd-P confirmed the phase purity and excellent stability in H_2_O and common organic solvents, as well as in metal salt aqueous system by matching the simulated pattern (Fig. S3†). The samples were heated for 8 h at different temperatures and the corresponding PXRD diagrams were determined. As shown in Fig. S4,† when heated to 180 °C, Cd-P framework still contains excellent chemical stability. High stability of Cd-P may result from the formation of strong bonds between N/O atoms and Cd^2+^ ions based on Pearson's hard/soft acid/base principle,^39,40^ or relatively highly dense 2D framework, and the synergy effect of these factors.^41^ Obviously, Cd-P framework with excellent chemical stability offers the possibility for its practical application as a metal ion sensor.

Afterward, Cd-P was soaked in the aqueous solution of Tb(NO_3_)_3_ to obtain Tb^3+^@Cd-P. The crystalline integrity of Cd-P remained unchanged after the incorporation of Tb^3+^ ion, which was confirmed by PXRD. In order to explore the stability of the Tb^3+^@Cd-P, the sample was treated with FeCl_3_ solution. After multiple immersion in FeCl_3_ solution for 12 h, the PXRD patterns of Tb^3+^@Cd-P showed the excellent stability of the crystalline integrity (Fig. S5†). To obtain the number of Tb^3+^ ions doping into Cd-P, EDS analysis on Tb^3+^@Cd-P was performed (Fig. S6†). The result showed that the ratio of Cd^2+^ and Tb^3+^ ions was approximately 5 : 1. Further, X-ray photoelectron spectroscopy (XPS) analyses on Tb^3+^@Cd-P and Cd-P were performed. As shown in Fig. S7,† after treatment by Tb^3+^, three new peaks at 1277.6, 1243.4 and 151 eV appeared corresponding to Tb 3d_3/2_ Tb 3d_5/2_, Tb 4d, by which the existence of Tb^3+^ ions in the composites can be ascertained.^42^

### 3.3. Fluorescence properties

Considering that CPs composed of d^10^ metal ions and aromatic organic ligands may be the promising luminescent materials,^43^ the fluorescence properties of Cd-P were investigated in 5 mM Tris–HCl/NaCl buffer (pH 7.0) at room temperature. Under 318 and 328 nm excitation, the luminescence of Cd-P and Tb^3+^@Cd-P in aqueous solution show the intense emission centered at 400 and 390 nm, respectively, while the luminescence of modbc exhibits a similar emission centered at 475 nm at 412 nm excitation (Fig. S8†). Moreover, the fluorescence emission spectra of Cd-P and Tb^3+^@Cd-P show good fluorescence stability within 12 h. In addition, the fluorescence intensity of Cd-P and Tb^3+^@Cd-P dispersed in aqueous solution is basically unchanged in the range of pH 0.5–14.0 (Fig. S9†). Obviously, Cd-P framework with excellent chemical stability offers the possibility for its practical application based on the fact that industrial effluent and polluted rivers are usually acidic or alkaline.^44^

### 3.4. Detection of ions

**Fig. 2:**
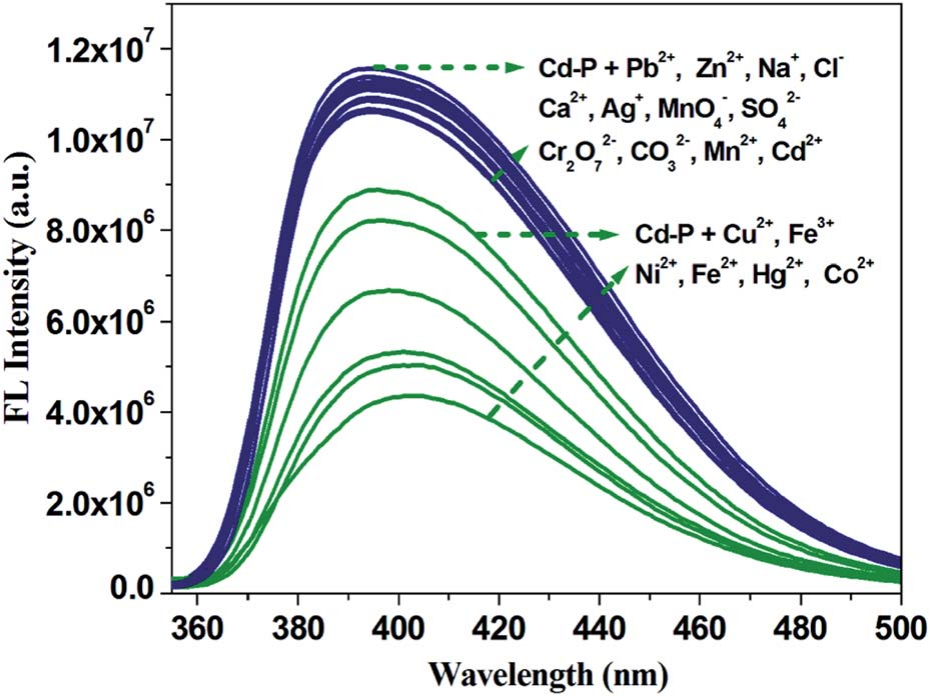
Luminous intensity of Cd-P upon different ions at 400 nm in 5 mM Tris–HCl/NaCl buffer (pH 7.0). [Cd-P] = 1.0 × 10^−5^ M and [ions] = 2.5 μM. *λ*_ex_: 318 nm, *λ*_F_: 400 nm, slit width: 4 nm.

To detect water pollution, we explored the potential detection of Cd-P to various cations and anions. The same concentrations (1.0 × 10^−3^ M) of aqueous solutions of Na^+^, Ag^+^, Cd^2+^, Cu^2+^, Zn^2+^, Pb^2+^, Ca^2+^, Mn^2+^, Co^2+^, Ni^2+^, Fe^2+^, Hg^2+^, Fe^3+^, MnO_4_^−^, CO_3_^2−^, SO_4_^2−^, Cl^−^, and Cr_2_O_7_^2−^ were prepared, and we investigated their effects on the fluorescence intensity of Cd-P. As shown in Fig. 2, Cd-P shows selective sensing ability toward different cations and anions. Obviously, Cu^2+^, Co^2+^, Ni^2+^, Fe^2+^, Hg^2+^ and Fe^3+^ ions present the higher quenching efficiency.

**Fig. 3:**
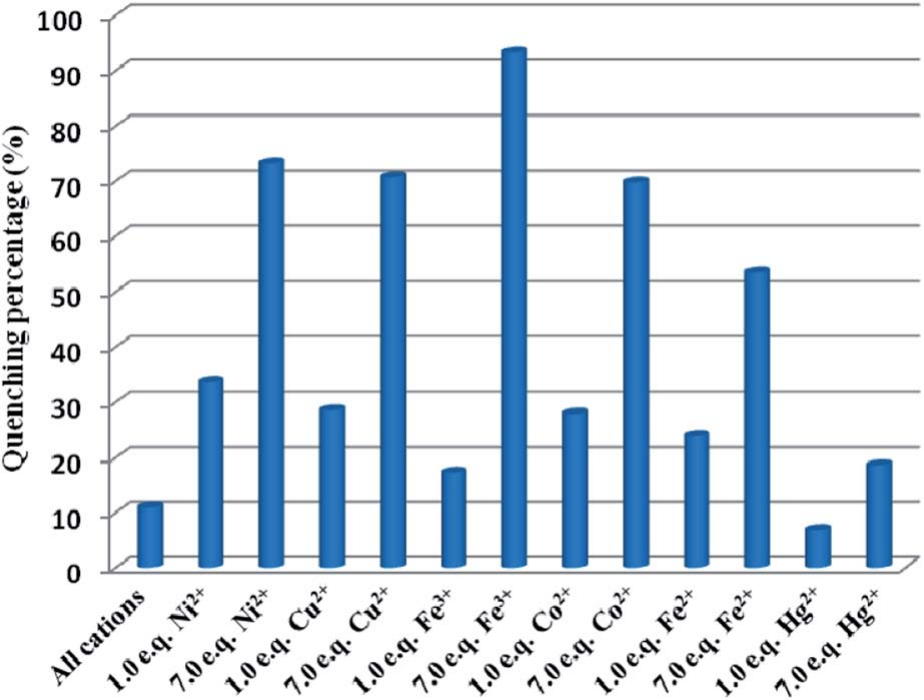
Comparison of the luminescence intensity of Cd-P in 5 mM Tris–HCl/NaCl buffer (pH 7.0): blank, after addition of mixed ions (Na^+^, Ag^+^, Cd^2+^, Zn^2+^, Pb^2+^, Ca^2+^, Mn^2+^; total concentration of mixed metal ions is 35 μM), and followed by addition of Cu^2+^, Co^2+^, Ni^2+^, Fe^2+^, Hg^2+^ and Fe^3+^ ions, respectively ([analyte] = 5 or 35 μM). *λ*_ex_: 318 nm, *λ*_F_: 400 nm, slit width: 4 nm.

To investigate the fluorescence sensitivity ability of Cd-P for detecting Cu^2+^, Co^2+^, Fe^2+^, Hg^2+^, Ni^2+^ and Fe^3+^ ions, the corresponding luminescence spectra were recorded by ion concentration titration. As shown in Fig. S10,† the remarkable fluorescence declines of Cd-P are observed in ionic concentration range. Furthermore, based on the application requirements of wastewater or pollutant detection, the anti-interference ability of Cd-P sensing for Cu^2+^, Co^2+^, Fe^2+^, Hg^2+^, Ni^2+^ and Fe^3+^ ions to other metal ions was explored at the same fluorescence measurement conditions. It is quite pleasing that the quenching effects by Cu^2+^, Co^2+^, Fe^2+^, Hg^2+^, Ni^2+^ and Fe^3+^ ions on the luminescence intensity of Cd-P are almost not influenced by the interfering metal ions, as shown in Fig. 3 and S11.†

**Fig. 4:**
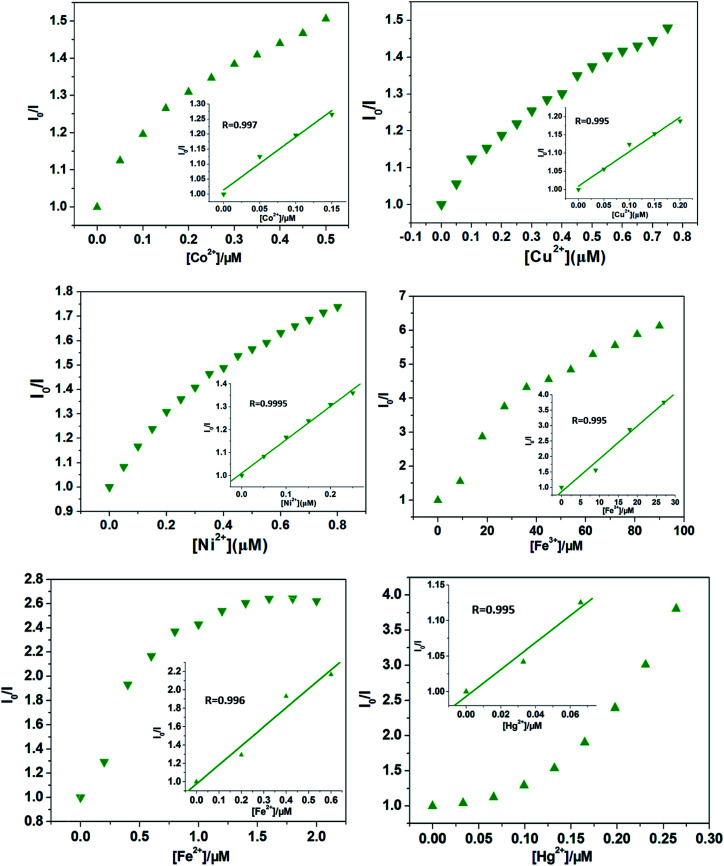
The Stern–Volmer plot of *I*_0_/*I versus* Co^2+^, Ni^2+^, Fe^3+^, Fe^2+^, Hg^2+^, and Cu^2+^ ions concentration, respectively (insets: the related Stern–Volmer plots at low Co^2+^, Ni^2+^, and Cu^2+^ ions concentration). *λ*_ex_: 318 nm, *λ*_F_: 400 nm, slit width: 4 nm.

Quantitatively, at low concentrations, the good linearity plots of Co^2+^, Ni^2+^ Fe^3+^, Fe^2+^, Hg^2+^ and Cu^2+^ ions were obtained, as shown in Fig. 4. Also, the corresponding quenching constants, *K*_sv_, were calculated, 1.76 ± 0.18 × 10^6^ M^−1^ for Co^2+^ ions, 0.95 ± 0.89 × 10^6^ M^−1^ for Cu^2+^ ions, 1.46 ± 0.058 × 10^6^ M^−1^ for Ni^2+^ ions, 1.06 ± 0.11 × 10^5^ M^−1^ for Fe^3+^ ions, 2.07 ± 0.27 × 10^6^ M^−1^ for Fe^2+^ ions, and 1.89 ± 0.36 × 10^6^ M^−1^ for Hg^2+^ ions, respectively. In addition, the LODs based on Cd-P were also obtained and 0.19 ± 0.06 μM for Co^2+^ ions, 0.26 ± 0.04 μM for Cu^2+^ ions, 0.23 ± 0.03 μM for Ni^2+^ ions, 0.047 ± 0.002 μM for Fe^3+^ ions, 0.58 ± 0.013 nM for Fe^2+^ ions, and 5.79 ± 2.0 mM for Hg^2+^ ions, respectively. However, at higher concentrations, the Stern–Volmer figures deviate from the line maybe due to energy transfer processes or self-absorption.^37^ In this work, the calculated LOD values of Cd-P for Cu^2+^, Ni^2+^ and Fe^3+^ ions are far lower than maximum allowable levels (MAL) in drinking water as required by the Environmental Protection Agency (EPA, 15.7, 0.34, and 5.36 μM, respectively).^45^

### 3.5. Differentiation of Co^2+^, Ni^2+^, Fe^3+^, Fe^2+^, Hg^2+^ and Cu^2+^ ions

**Fig. 5:**
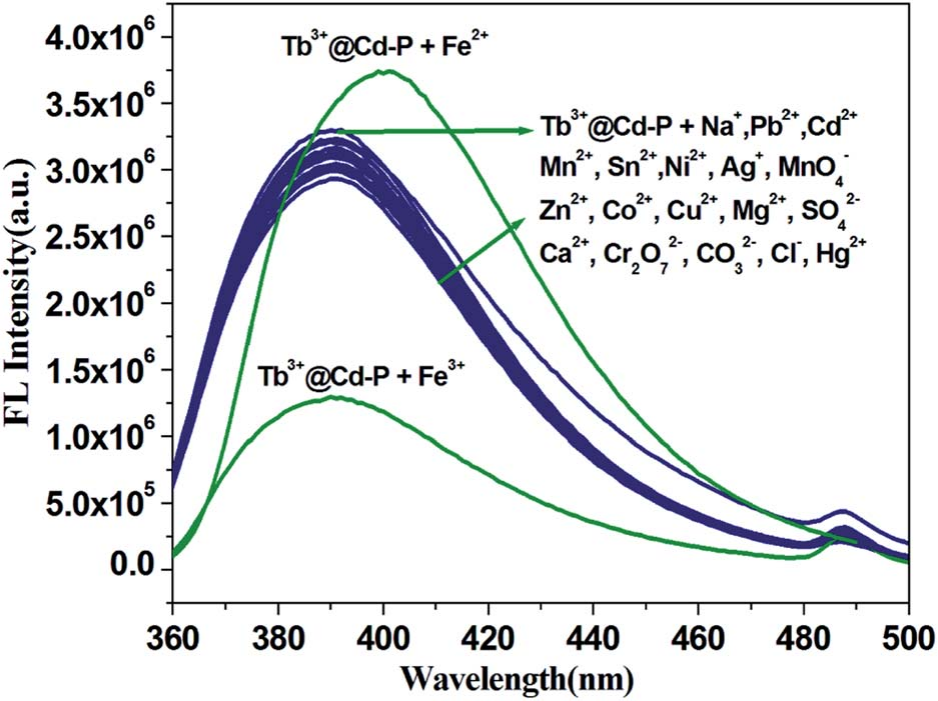
Luminous intensity of Tb^3+^@Cd-P upon different ions at 390 nm in 5 mM Tris–HCl/NaCl buffer (pH 7.0). [Tb^3+^@Cd-P] = 1.0 × 10^−5^ M and [ions] = 2.5 μM. *λ*_ex_: 328 nm, *λ*_F_: 390 nm, slit width: 4 nm.

It is worth noting that Cd-P cannot selectively distinguish Cu^2+^, Co^2+^, Ni^2+^, Fe^2+^, Hg^2+^ and Fe^3+^ ions. To improve the selectivity of Cd-P, Tb^3+^@Cd-P was obtained *via* PSM. As expected, with the concentration titration of Fe^3+^ ions, the luminescence intensity of Tb^3+^@Cd-P is quenched prominently (Fig. S12†). Further, we investigated the selectivity of Tb^3+^@Cd-P toward a wide range of ions. The other measured ions show a negligible influence to the emission intensity of Tb^3+^@Cd-P except Fe^3+^ and Fe^2+^ ions, as shown in Fig. 5. To examine the sensing behavior of Tb^3+^@Cd-P to Fe^2+^, titration experiment of Tb^3+^@Cd-P with Fe^2+^ was performed (Fig. S13†). On gradual addition of Fe^2+^, the fluorescence of Tb^3+^@Cd-P solutions significantly enhanced. We also observed that (NH_4_)_2_Fe(SO_4_)_2_ exhibited the fluorescence emission at about 390 nm, as deposited in Fig. S14.† Therefore, we speculate that the luminescence of (NH_4_)_2_Fe(SO_4_)_2_ is responsible for the fluorescence enhancement by Fe^2+^. These results indicate that Tb^3+^@Cd-P can detect Fe^3+^ selectively among coexisting ions.

**Fig. 6:**
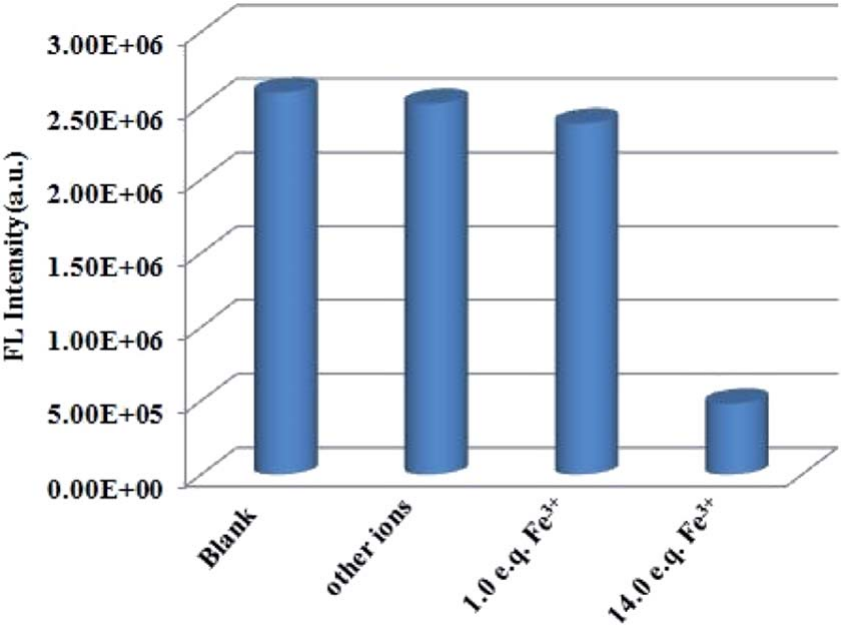
Comparison of the luminescence intensity of Tb^3+^@Cd-P in 5 mM Tris–HCl/NaCl buffer (pH 7.0): after addition of mixed ions (Na^+^, Ag^+^, Cd^2+^, Zn^2+^, Pb^2+^, Ca^2+^, Cu^2+^, Co^2+^, Ni^2+^, Sn^2+^, Mg^2+^, Hg^2+^, Fe^2+^, Mn^2+^; total concentration of mixed metal ions is 70 μM), and followed by addition of Fe^3+^ ions ([Fe^3+^] = 5 or 70 μM). *λ*_ex_: 328 nm, *λ*_F_: 390 nm, slit width: 4 nm.

To further evaluate the anti-interference ability of Tb^3+^@Cd-P as a selective sensor for Fe^3+^, competitive experiments were conducted in the presence of other metal ions (Fig. 6 and S15†).^46^ No significant differences in luminescence intensity can be observed among these solutions. All these results clearly indicate Tb^3+^@Cd-P has a high selectivity and anti-interference ability in the detection of Fe^3+^ under aqueous conditions.^47^

**Fig. 7:**
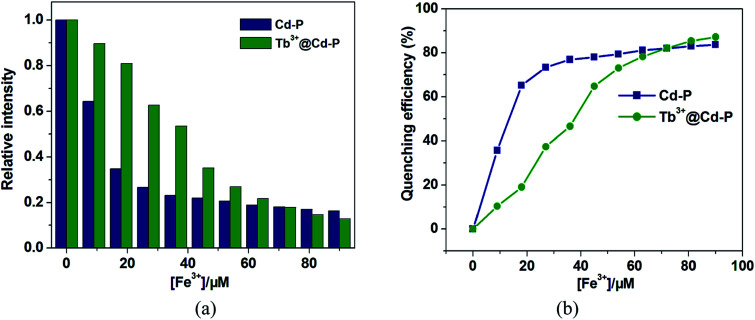
(a) Relative fluorescence intensity and (b) the corresponding quenching efficiency of Cd-P and Tb^3+^@Cd-P *vs.* different Fe^3+^ ion concentrations. *λ*_ex_: 328 nm, *λ*_F_: 390 nm for Tb^3+^@Cd-P, slit width: 4 nm; *λ*_ex_: 318 nm, *λ*_F_: 400 nm for Cd-P, slit width: 4 nm.

One unexpected finding is that both Cd-P and Tb^3+^@Cd-P are easily sensitized by Fe^3+^ ions, and the rapid response time of the sensitization process obtained by the experiment is approximately 10 s (Fig. S16†). Compared with Cd-P, the influence of Fe^3+^ ions on the luminescence intensity of Tb^3+^@Cd-P is significantly smaller (Fig. 7a and b). To better analyze and compare with Cd-P, the Stern–Volmer plot of Tb^3+^@Cd-P for detecting Fe^3+^ was obtained (Fig. S17†), a linear presented at lower and higher experimental concentration range (0–20 μM; 36–90 μM), and the *K*_SV_ and LOD values were 1.31 ± 0.01 × 10^4^ M^−1^, 1.09 ± 0.02 × 10^5^ M^−1^, respectively, displaying the high quenching efficiency of the Fe^3+^ ions.^48–50^ In addition, the LODs based on Tb^3+^@Cd-P were also obtained and 0.66 ± 0.02 μM for Fe^3+^ ions.

For Fe^3+^ ions at lower concentration, the values of *K*_sv_[Tb^3+^@Cd-P]/*K*_sv_[Cd-P] and LOD[Tb^3+^@Cd-P]/LOD[Cd-P] were 0.12 and 14.04, respectively, which further verify the above conclusion. In short, although Cu^2+^, Co^2+^, Fe^2+^, Hg^2+^, Ni^2+^ and Fe^3+^ ions can reduce the luminescence intensity of Cd-P, the luminescence quenching of Tb^3+^@Cd-P is only affected by Fe^3+^ ions. In addition, the luminescence quenching degree of Tb^3+^@Cd-P and Cd-P for Fe^3+^ ions is also different. We can selectively differentiate Fe^3+^ ions by combining the changes of the luminescence intensities of Tb^3+^@Cd-P and Cd-P. It exhibits a better performance, and the LODs were estimated to be 10^−7^ M, which also lies well below several MOF fluorescent sensors for detecting Fe^3+^ ion for a specific comparison (Table S3†).

**Fig. 8:**
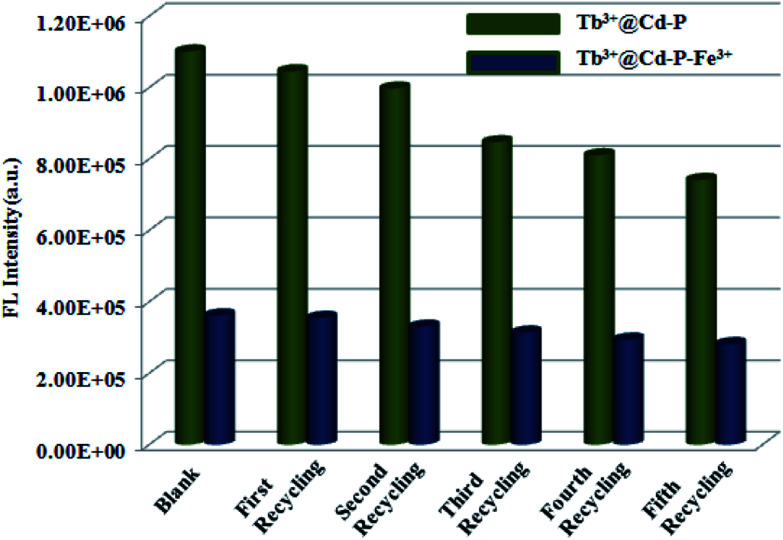
Five regeneration cycles for detection of Fe^3+^ ions by Tb^3+^@Cd-P. *λ*_ex_: 328 nm, *λ*_F_: 390 nm, slit width: 4 nm.

Considering the cost of emitting probes, their regenerative properties play an important role in practical applications. Hence, to obtain Tb^3+^@Cd-P + Fe^3+^ samples, we dispersed Tb^3+^@Cd-P in the aqueous solution of Fe(NO_3_)_3_ (10^−3^ M) for 12 h, and separated by centrifugation then washed with deionized water to remove the remaining Fe^3+^ ions. The emission intensity of recovered Tb^3+^@Cd-P is well comparable to that of the parent sample (Fig. 8 and S18†). Fortunately, after five regeneration cycles, the yield of Tb^3+^@Cd-P to differentiate Fe^3+^ ions reaches about 78%. Above results confirm that Tb^3+^@Cd-P can achieve a differential detection toward Cu^2+^, Co^2+^, Ni^2+^ and Fe^3+^ ions, and exhibits high detection sensitivities. Moreover, as an Fe^3+^ ion-responsive probe, the Tb^3+^@Cd-P can exert the anti-interference, regenerative recognition and fast procedure under aqueous solution.

### 3.6. Discussion of the mechanism

The high stabilities of Cd-P (Fig. S3 and S4†) and Tb^3+^@Cd-P (Fig. S5†) in aqueous solution of metal salts, organic solvents, different temperatures, and the solutions with pH range from 0.5 to 14.0 (Fig. S9†) suggest that the luminescence quenching is not caused by framework collapse. In order to observe whether these metal ions can enter the frame structure of Cd-P, ICP-AES analysis on Cd-P has been performed, as shown in Table S4.† Further, the XPS spectra of Cd-P before and after added Cu^2+^, Co^2+^, Ni^2+^, Fe^3+^ metal salt samples present that both carboxyl oxygen atoms of Cd-P and hydroxyl oxygen atoms of H_2_O participate in the coordination of Fe^3+^*etc* metal ions (Fig. S19†).^28^

**Fig. 9:**
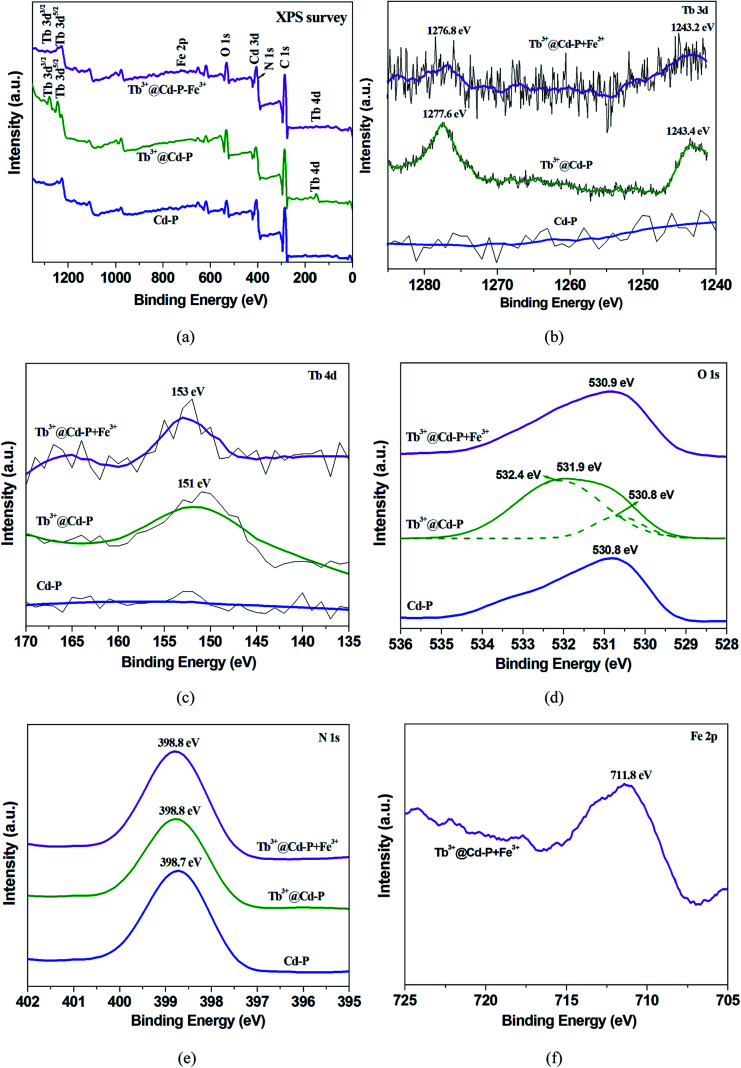
The XPS spectra of Cd-P, Tb^3+^@Cd-P, and Tb^3+^@Cd-P + Fe^3+^ samples: (a) the survey spectrum, the high resolution XPS spectra of (b) Tb 3d, (c) Tb 4d, (d) O 1s, (e) N 1s, and (f) Fe 2p, respectively.

After Cd-P treatment by Tb^3+^ ions, EDS analysis on Tb^3+^@Cd-P shows that the ratio of Cd^2+^ and Tb^3+^ ions is approximately 5 : 1 (Fig. S6†). Fig. S20† shows that the UV-vis absorption band of Tb^3+^@Cd-P presents a significant red shift compared to Cd-P, indicating that Tb^3+^ ions interacts with Cd-P.^51^ Further, XPS analyses on Cd-P, Tb^3+^@Cd-P and Tb^3+^@Cd-P + Fe^3+^ also show that the luminescent CPs are high stability, and the luminescence quenching is not caused by framework collapse (Fig. S21† and 9a). Three new peaks at 1277.6, 1243.4 and 151 eV appear corresponding to Tb 3d_3/2_ Tb 3d_5/2_, Tb 4d, by which the existence of Tb^3+^ ions in the composites of Tb^3+^@Cd-P can be ascertained (Fig. 9b and c).^42^ The O 1s spectrum of Tb^3+^@Cd-P shown in Fig. 9d can be fitted into two peaks at 530.8 eV and 532.4 eV, which correspond to carboxyl group oxygen atoms and –OH species, respectively. In addition, the binding energy of N 1s does unchange (Fig. 9e), implying that the N atom of modbc may not be involved in the coordination interaction with Tb^3+^ ions. The result shows that carboxyl oxygen atoms of Cd-P and hydroxyl oxygen atoms of H_2_O participate in the coordination of Tb^3+^ ions.^28^ Therefore, the emission peak of Tb^3+^@Cd-P shows a significant blue shift from 400 to 390 nm after post-treatment by Tb^3+^ ions, which can be attributed to the O atom of modbc coordinated with Tb^3+^ ions, but does not result in the “antenna effect” obtained from the 4f–4f electron transitions.^25^ Tb^3+^@Cd-P after treatment by Fe^3+^ ions, the corresponding Fe 2p peak is found supporting the presence of Fe in Tb^3+^@Cd-P (Fig. 9f). The O 1s peak further shifts to 530.9 eV (Fig. 9d). The fast and simple regeneration method also shows that the binding between Tb^3+^@Cd-P and Fe^3+^ ions should be weaker (Fig. 8 and S18†), which may be that Fe^3+^ ions spread to the frameworks of CPs leading to luminescence quenching.^7^

In addition, Fig. S8 and S20† show that there exists extensive overlap between UV-vis absorbance of Fe^3+^ and the excitation absorbance of Tb^3+^@Cd-P, while negligible for other metal ions.^52^ As well as the absorbance of Fe^3+^ corresponding to the excitation absorbance of Tb^3+^@Cd-P, ultimately lead to the luminescence quenching of Tb^3+^@Cd-P.^53,54^ As shown in Fig. S22 and Table S5,† the lifetimes are shortened from 4.15 to 1.99 ns, 4.15 to 1.94 ns, and 4.15 to 1.98 ns after Cd-P treated with Cu^2+^, Co^2+^, and Ni^2+^ ions, and the downward Stern−Volmer curve at higher Cu^2+^, Co^2+^ or Ni^2+^ ion concentrations demonstrate that both static and dynamic mechanisms operate at higher concentration. However, the lifetimes of Cd-P and Tb^3+^@Cd-P remain in the presence and absence of Fe^3+^ ions (Fig. S22, S23 and Table S4†), and the Stern–Volmer plots are linear at the full experimental concentration region, which suggest that there are the static quenching mechanisms in selectively differentiate Fe^3+^ ions by combining Tb^3+^@Cd-P and Cd-P.^55^

## 4. Conclusion

In summary, a Cd(ii)-based polymer framework (Cd-P) was constructed and exhibited high sensitivity (*K*_sv_ ∼10^6^ M^−1^ and LODs ∼10^−7^ M) toward Cu^2+^, Co^2+^, Hg^2+^, Fe^2+^, Ni^2+^ and Fe^3+^ ions *via* luminescence quenching. In addition, a fluorescent hybrid material, Tb^3+^@Cd-P, derived from Cd-P *via* PSM, which maintains high chemical stability, good selectivity, and excellent response toward Fe^3+^ ions, is most likely associated with the competition between the excitation of Tb^3+^@Cd-P and the absorption of Fe^3+^ ions, wherein there is also the weak interaction of Tb^3+^@Cd-P with Fe^3+^ ions. Also, we can differentiate selectivity Fe^3+^ ions by the combination of Cd-P and Tb^3+^@Cd-P. It should be mentioned that the two-dimensional luminescent probes for detecting a trace amount of Fe^3+^ ions (μM) and quick response time (∼10 s) are still few reports. Most importantly, Tb^3+^@Cd-P can act as a reversible luminescent probe for Fe^3+^ ions with potential application.

## Conflicts of interest

The authors declare no competing financial interest.

## Supplementary Material

RA-009-C9RA06639J-s001

RA-009-C9RA06639J-s002

## References

[cit1] Cao X. H., Zhao N., Gao A. P., Ding Q. Q., Li Y. R., Chang X. P. (2018). Langmuir.

[cit2] Panja A., Ghosh K. (2018). Mater. Chem. Front..

[cit3] Mako T. L., Racicot J. M., Levine M. (2019). Chem. Rev..

[cit4] Wang B., Lv X. L., Feng D., Xie L. H., Zhang J., Li M., Xie Y. B., Li J. R., Zhou H. C. (2016). J. Am. Chem. Soc..

[cit5] Tian D., Li Y., Chen R. Y., Chang Z., Wang G. Y., Bu X. H. (2014). J. Mater. Chem. A.

[cit6] Kreno L. E., Leong K., Farha O. K., Allendorf M., Van Duyne R. P., Hupp J. T. (2012). Chem. Rev..

[cit7] Yan W., Zhang C. L., Chen S. G., Han L. J., Zheng H. (2017). ACS Appl. Mater. Interfaces.

[cit8] Ji G. F., Liu J. J., Gao X. C., Sun W., Wang J. Z., Zhao S. L., Liu Z. L. (2017). J. Mater. Chem. A.

[cit9] Xu X. Y., Yan B. (2016). Sens. Actuators, B.

[cit10] Zhang M., Feng G., Song Z. G., Zhou Y. P., Chao H. Y., Yuan D. Q., Tan T. T. Y., Guo Z. G., Hu Z. G., Tang B. Z., Liu B., Zhao D. (2014). J. Am. Chem. Soc..

[cit11] Bhattacharyya S., Chakraborty A., Jayaramulu K., Hazra A., Maji T. K. A. (2014). Chem. Commun..

[cit12] Du P. Y., Gu W., Liu X. (2016). Inorg. Chem..

[cit13] Moon S. Y., Cha N. R., Kim Y. H., Chang S. K. (2004). J. Org. Chem..

[cit14] Matsumiya H., Iki N., Miyano S. T. (2004). Anal. Bioanal. Chem..

[cit15] Liu X., Theil E. C. (2005). Acc. Chem. Res..

[cit16] Hyman L., Franz K. (2012). Coord. Chem. Rev..

[cit17] Bricks J. L., Kovalchuk A., Trieflinger C., Nofz M., Büschel M., Tolmachev A. I., Daub J., Rurack K. (2005). J. Am. Chem. Soc..

[cit18] Zhang S. R., Du D. Y., Qin J. S., Bao S. J., Li S. L., He W. W., Su Z. M. (2014). Chem.–Eur. J..

[cit19] Liu T. F., Feng D. W., Chen Y. P., Zou L. F., Bosch M., Yuan S., Wei Z. W., Fordham S., Wang K. C., Zhou H. C. (2015). J. Am. Chem. Soc..

[cit20] Yi F., Chen D., Wu M., Han L., Jiang H. (2016). ChemPlusChem.

[cit21] Hu Z., Deibert B. J., Li J. (2014). Chem. Soc. Rev..

[cit22] Lustig W. P., Mukherjee S., Rudd N. D., Desai A. V., Li J., Ghosh S. K. (2017). Chem. Soc. Rev..

[cit23] Xu X., Yan B. (2015). ACS Appl. Mater. Interfaces.

[cit24] Rao P. C., Mandal S. (2018). Inorg. Chem..

[cit25] Ji G., Wang J., Gao X., Liu J., Guan W., Liu H., Liu Z. (2018). Eur. J. Inorg. Chem..

[cit26] Lin Z. J., Lu J., Hong M., Cao R. (2014). Chem. Soc. Rev..

[cit27] Ji G. F., Gao X. C., Zheng T. X., Guan W. H., Liu H. T., Liu Z. L. (2018). Inorg. Chem..

[cit28] Hao J. N., Yan B. (2016). Nanoscale.

[cit29] An J., Shade C. M., Czegan D. A. C., Petoud S., Rosi N. L. (2011). J. Am. Chem. Soc..

[cit30] Buschbaum K. M., Beuerle F., Feldmann C. (2015). Microporous Mesoporous Mater..

[cit31] Meyer L. V., Schonfeld F., Muller-Buschbaum K. (2014). Chem. Commun..

[cit32] Zhang Z., He Y., Liu L., Lu X., Zhu X., Wong W., Pan M., Su C. (2016). Chem. Commun..

[cit33] Xia T., Cui Y., Yang Y., Qian G. (2016). ChemNanoMat.

[cit34] Lv R., Li H., Su J., Fu X., Yang B. Y., Gu W., Liu X. (2017). Inorg. Chem..

[cit35] Basudeb D., Rajkumar J., Anup K. B., Partha P. R., Chittaranjan S., Mohammad H. M. (2019). Inorg. Chem..

[cit36] Mürsel A. (2017). Cryst. Growth Des..

[cit37] Hou Y. L., Xu H., Cheng R. R., Zhao B. (2015). Chem. Commun..

[cit38] Li L., Shen S., Lin R., Bai Y., Liu H. (2017). Chem. Commun..

[cit39] Yi F. Y., Wang S. C., Gu M., Zheng J. Q., Han L. (2018). J. Mater. Chem. C.

[cit40] Yao Z. Q., Li G. Y., Xu J., Hu T. L., Bu X. H. (2018). Chem.–Eur. J..

[cit41] Yu C., Shao Z., Hou H. (2017). Chem. Sci..

[cit42] Hao J. N., Yan B. (2017). Adv. Funct. Mater..

[cit43] Liu C., Yan B. (2015). Photochem. Photobiol. Sci..

[cit44] Liang L. F., Liu L. Y., Jiang F. L., Liu C. P., Yuan D. Q., Chen Q. H., Wu D., Jiang H. L., Hong M. C. (2018). Inorg. Chem..

[cit45] WHO , WHO Guidelines for Drinking-Water Quality, 4th edn, WHO Press, Geneva, 2011

[cit46] Zhang Z. Y., Lu S. Z., Sha C. M., Xu D. M. (2015). Sens. Actuators, B.

[cit47] Hu C. C., Gao Q., Zhu Z. X., Chang L. L., Zhou W. J., Xia K. S., Han B., Zhou C. G. (2018). Sens. Actuators, B.

[cit48] Wen G. X., Wu Y. P., Dong W. W., Zhao J., Li D. S., Zhang J. (2016). Inorg. Chem..

[cit49] Fan K., Bao S. S., Nie W. X., Liao C. H., Zheng L. M. (2018). Inorg. Chem..

[cit50] Wu Z. F., Gong L. K., Huang X. Y. (2017). Inorg. Chem..

[cit51] Ji G., Yang Z., Zhang H., Zhao Y., Yu B., Ma Z., Liu Z. (2016). Angew. Chem., Int. Ed..

[cit52] Shylaja A., Roja S. S., Priya R. V., Kumar R. R. (2018). J. Org. Chem..

[cit53] Wen G. X., Wu Y. P., Dong W. W., Zhao J., Li D. S., Zhang J. (2016). Inorg. Chem..

[cit54] Neupane L. N., Oh E. T., Park H. J., Lee K. H. (2016). Anal. Chem..

[cit55] Gole B., Bar A. K., Mukherjee P. S. (2014). Chem.–Eur. J..

