# Supplementary material for: Two-dimensional Cd(ii) coordination polymer encapsulated by Tb3+ as a reversible luminescent probe for Fe3+
Source: RSC Adv. 2019 Oct 29;9(60):34949–57. doi: 10.1039/c9ra06639j (PMC9074126; doi:10.1039/c9ra06639j)
Supplement: RA-009-C9RA06639J-s001 [file RA-009-C9RA06639J-s001.pdf]

Electronic Supplementary Material (ESI) for RSC Advances.
This journal is © The Royal Society of Chemistry 2019

Supporting Information

Two-dimensional Cd(II) coordination polymer encapsulated by Tb^{3+} as a reversible luminescent probe for Fe^{3+}

Yuandi Wu,[a] Meihua Lin,[a] Dongyang Liu,[a] Ming Liu,[a] Jing Qian,[a*,b,c]

Corresponding authors: a* qianjinger@aliyun.com (J. Qian)

a *College of Chemistry, Tianjin Normal University, Tianjin 300387, P. R. China*

b *Tianjin Key Laboratory of Structure and Performance for Functional Molecules, Tianjin Normal University, Tianjin 300387, P. R. China*

c *Key Laboratory of Inorganic−Organic Hybrid Functional Materials Chemistry, Tianjin Normal University, Ministry of Education, Tianjin 300387, P. R. China*

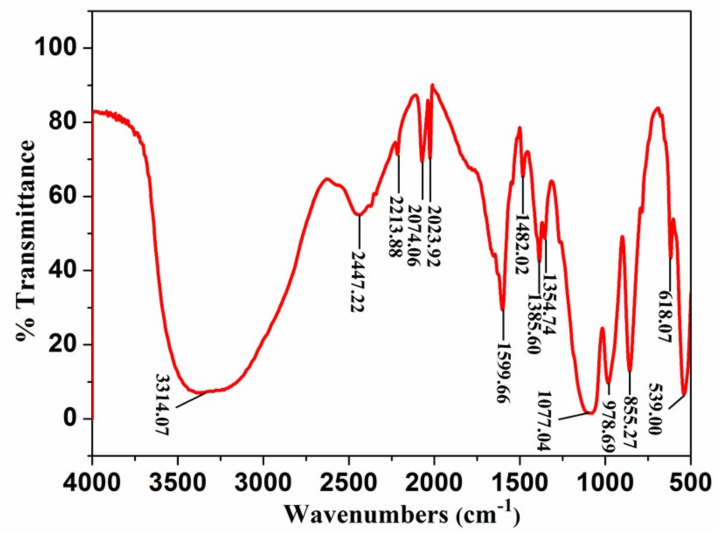

Fig. S1 FTIR patterns for Cd-P.

Table S1 Crystal data and structure refinement information for Cd-P.

polymer	Cd-P
CCDC	1881136
Formula	$Cd_{0.50}C_{12}H_8N_3O$
fw	266.41
temp (K)	296(2)
cryst syst	Tetragonal
Space group	P-4_32_12
a (Å)	10.7413(3)
b (Å)	10.7413(3)
c (Å)	20.7748(13)
$\alpha = \beta = \gamma$ (°)	90
V (A^3)	2396.9(2)
Z	8
ρ (Mg/m^3)	1.477
Abs coeff (mm^{-1})	0.943
F (000)	1064
GOF	1.069
R_1/wR_2 [I >2σ(I)]	0.0240/0.0620
R_1/wR_2 (all data)	0.0244/0.0623

Table S2 Selected bond lengths (A˚) and angles (◦) for Cd-P.

Bond lengths (Å)			
Cd(1)-N(1)#1	2.269(3)	Cd(1)-N(1)	2.269(3)
Cd(1)-N(2)#2	2.280(3)	Cd(1)-N(2)#3	2.280(3)
Cd(1)-O(1)#1	2.501(3)	Cd(1)-O(1)	2.501(3)
N(2)-Cd(1)#4	2.280(3)		
**Bond angles (º)**			
N(1)#1-Cd(1)-N(1)	101.05(18)	N(1)#1-Cd(1)-N(2)#2	141.02(11)
N(1)-Cd(1)-N(2)#2	95.08(14)	N(1)#1-Cd(1)-N(2)#3	95.08(14)
N(1)-Cd(1)-N(2)#3	141.02(11)	N(2)#2-Cd(1)-N(2)#3	94.16(19)
N(1)#1-Cd(1)-O(1)#1	55.88(10)	N(1)-Cd(1)-O(1)#1	127.90(11)
N(2)#2-Cd(1)-O(1)#1	86.29(10)	N(2)#3-Cd(1)-O(1)#1	90.43(11))
N(1)#1-Cd(1)-O(1)	127.90(11)	N(1)-Cd(1)-O(1)	55.88(10)
N(2)#2-Cd(1)-O(1)	90.43(11)	N(2)#3-Cd(1)-O(1)	86.29(10)
O(1)#1-Cd(1)-O(1)	175.18(14)		

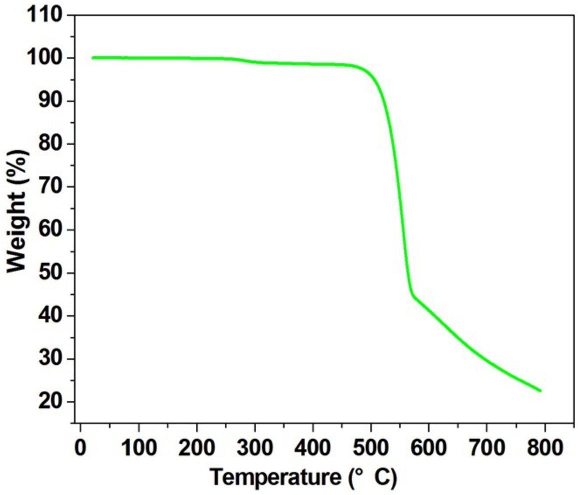

Fig. S2 Thermogravimetric curve of Cd-P.

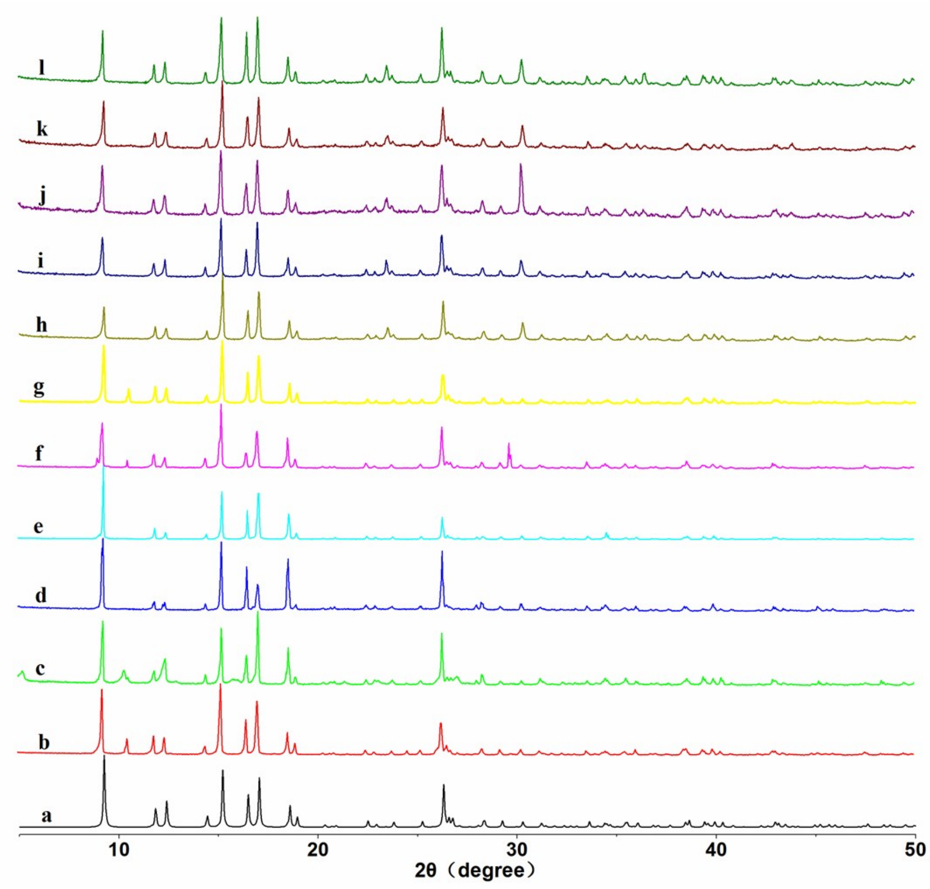

Fig. S3 PXRD patterns for Cd-P: (a) simulated; (b) experimental; (c) 3 days after immersion in water; (d) 3 days after immersion in $FeCl_3$ solution (e) 3 days after immersion in $CoCl_2$ solution; (f) 3 days after immersion in $NiCl_2$ solution; (g) 3 days after immersion in $CuCl_2$ solution; (h) 3 days after immersion in CH_3OH; (i) 3 days after immersion in C_2H_5OH; (j) 3 days after immersion in acetone; (k) 3 days after immersion in CH_2Cl_2; (l) 3 days after immersion in DMF.

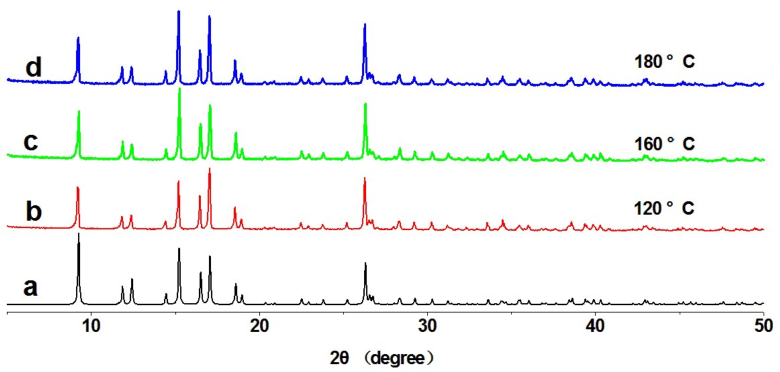

Fig. S4 PXRD patterns for Cd-P at different temperatures, 120℃ (red line); 160℃ (green line); 180℃ (blue line) and the simulated one calculated from the single crystal structure analysis (black line).

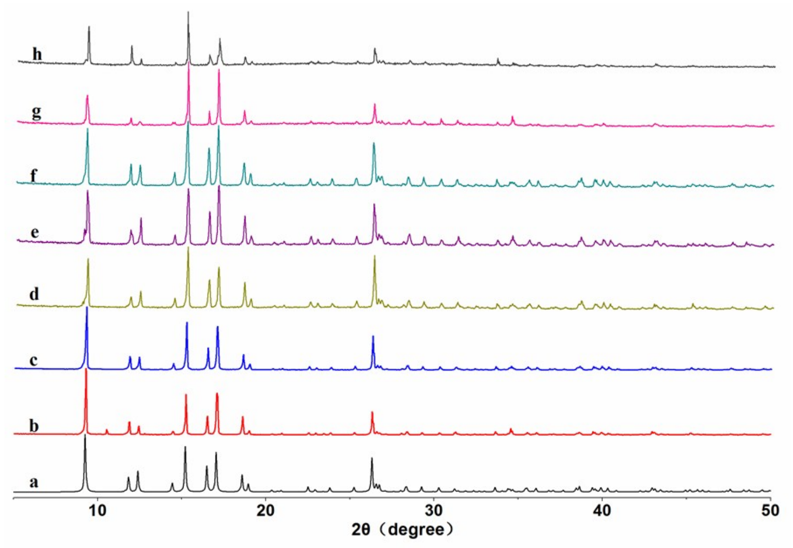

Fig. S5 PXRD patterns for Tb^{3+}@Cd-P: (a) simulated of Cd-P; (b) experimental of Cd-P; (c) experimental of Tb^{3+}@Cd-P; (d) experimental of Tb^{3+}@Cd-P first 12 h after immersion in $FeCl_3$ solution; (e) experimental of Tb^{3+}@Cd-P second 12 h after immersion in $FeCl_3$ solution; (f) experimental of Tb^{3+}@Cd-P third 12 h after immersion in $FeCl_3$ solution; (g) experimental of Tb^{3+}@Cd-P fourth 12 h after immersion in $FeCl_3$ solution; (h) experimental of Tb^{3+}@Cd-P fifth 12 h after immersion in $FeCl_3$ solution.

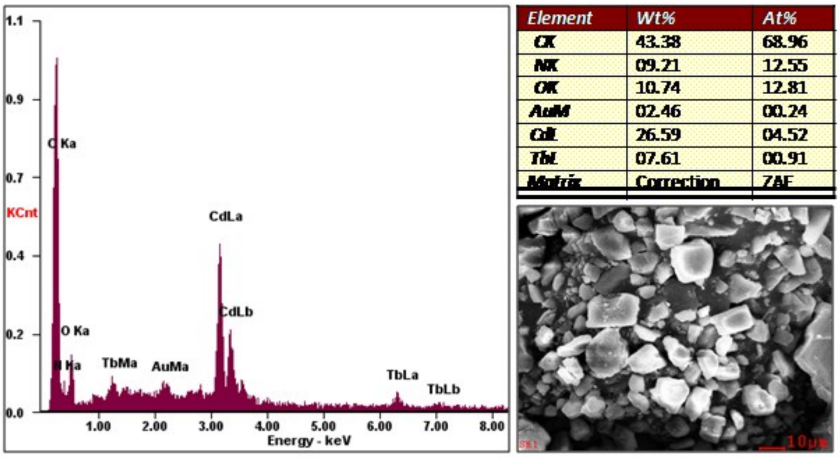

Fig. S6 SEM images and Elemental analysis by EDS of Tb^{3+}@Cd-P.

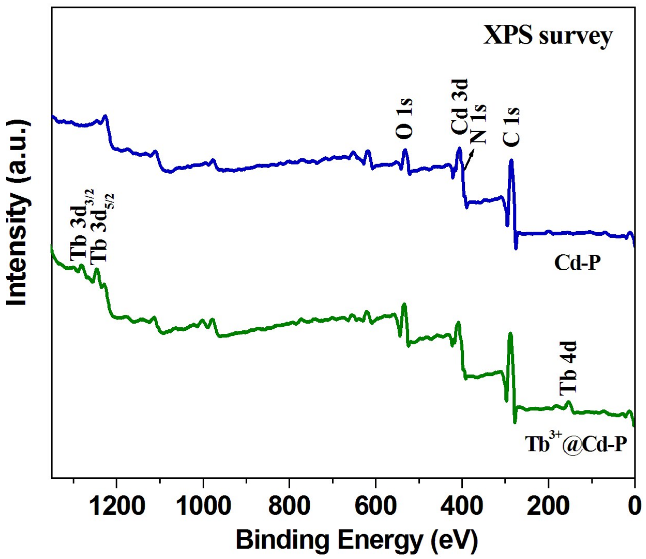

Fig. S7 XPS survey spectra of the Cd-P and Tb^{3+}@Cd-P samples.

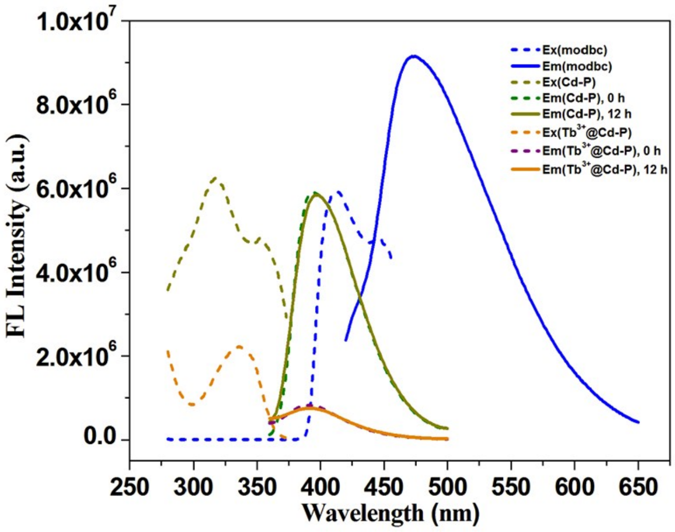

Fig. S8 liquid-state luminescent spectra of modbc ligand (blue), Cd-P (green and yellow) , Tb^{3+}@Cd-P (orange and purple) in 5 mM Tris-HCl/NaCl buffer (pH 7.0). slit width: 4 nm.

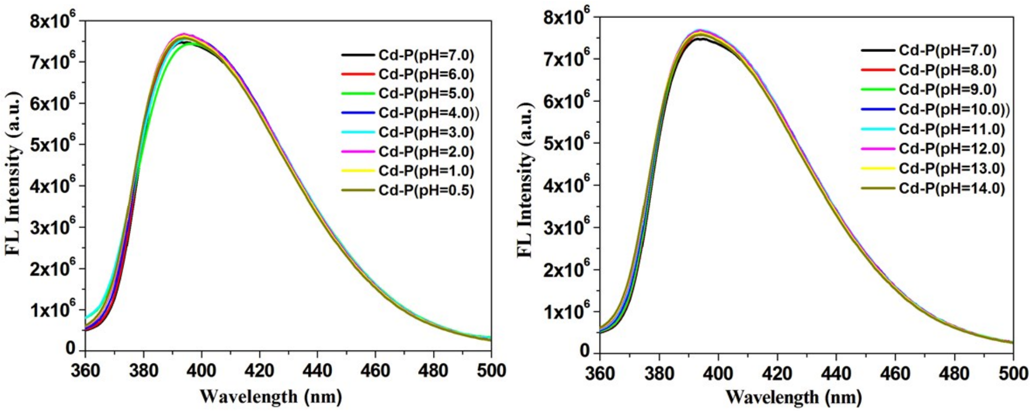

(a)

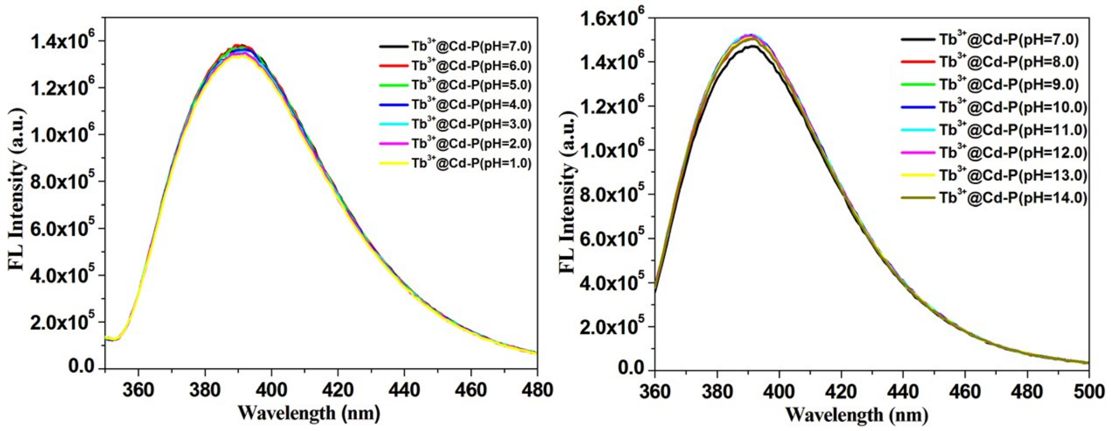

(b)

Fig. S9 (a) Liquid-state luminescent spectra of Cd-P with different pH values (0.5~14.0) in 5 mM Tris-HCl/NaCl buffer (pH 7.0). λ_{ex}: 318 nm, λ_F: 400 nm, slit width: 4 nm. (b) Tb^{3+}@Cd-P with different pH values (0.5~14.0) in 5 mM Tris-HCl/NaCl buffer (pH 7.0). λ_{ex}: 328 nm, λ_F: 390 nm, slit width: 4 nm.

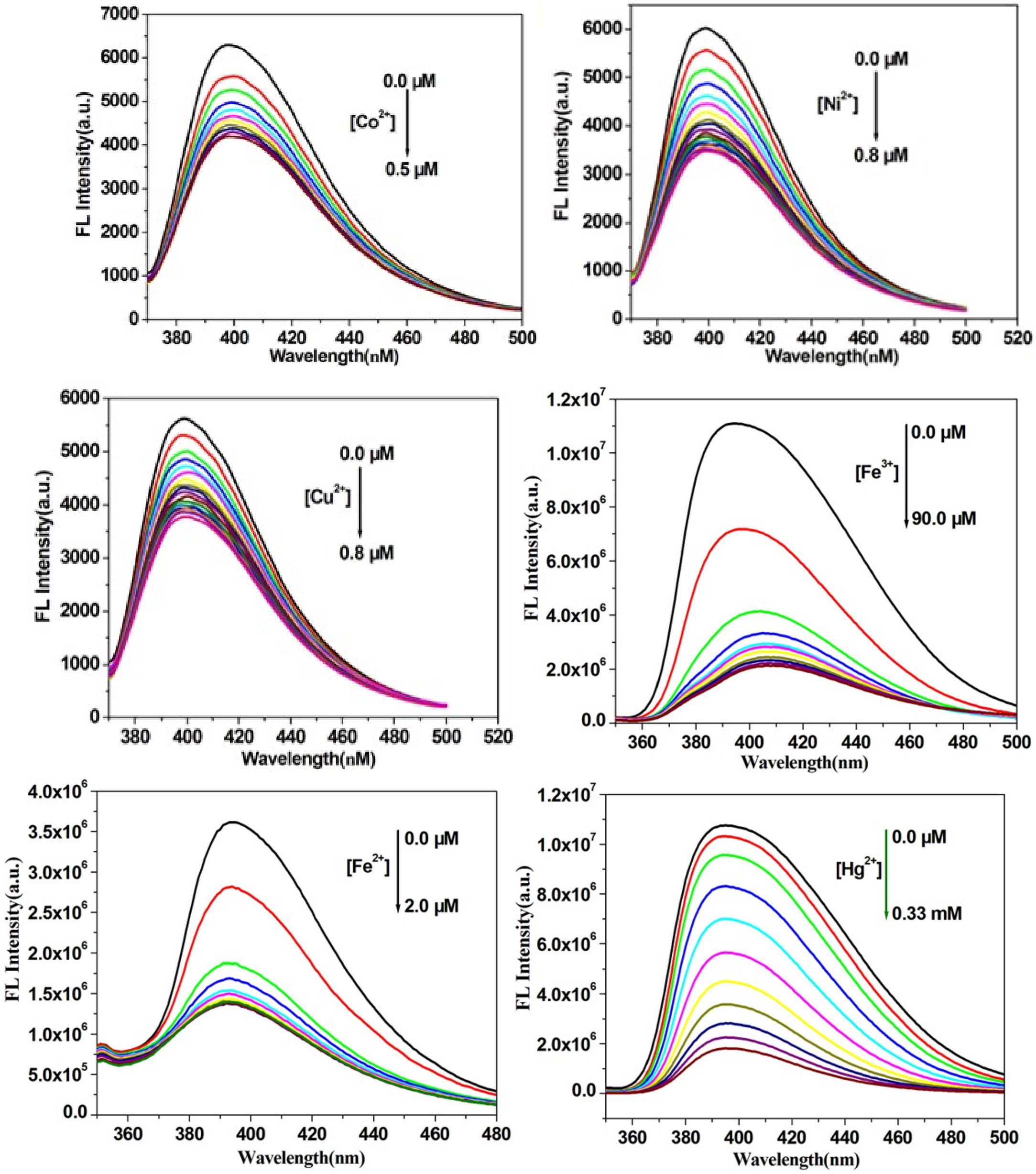

Fig. S10 Luminescence quenching of Cd-P in 5 mM Tris-HCl/NaCl buffer (pH 7.0) with gradual addition of 1 mM solution of Co^{2+}, Ni^{2+}, Fe^{3+}, Hg^{2+}, Fe^{2+} and Cu^{2+} ions, respectively. λ_{ex}: 318 nm, λ_F: 400 nm, slit width: 4 nm.

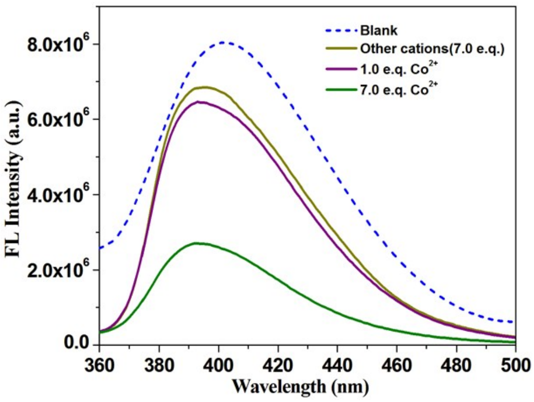

(a)

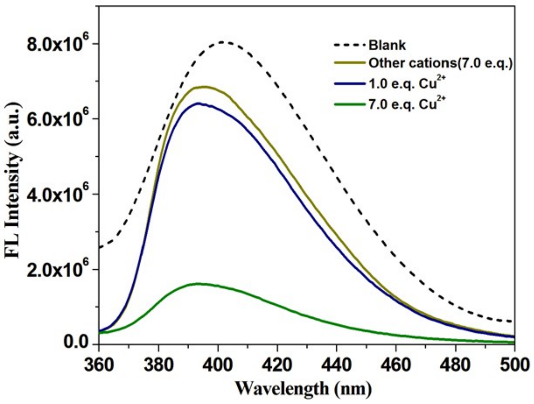

(b)

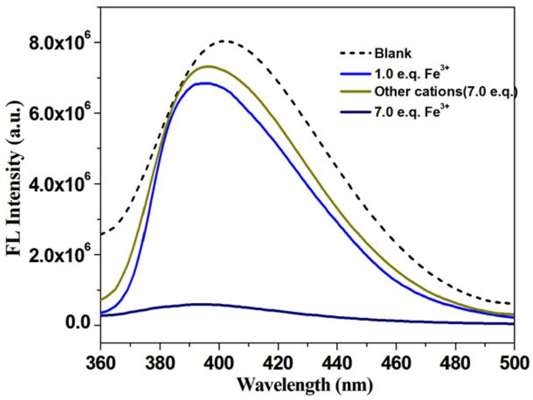

(c)

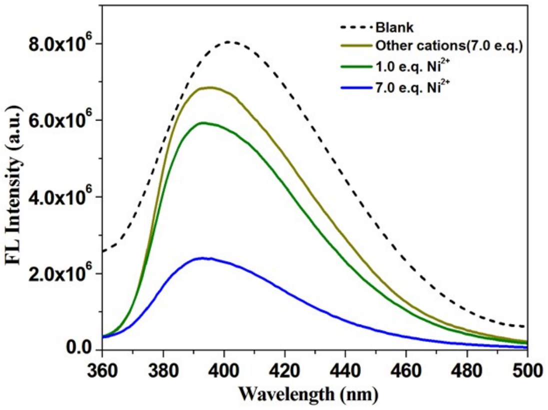

(d)

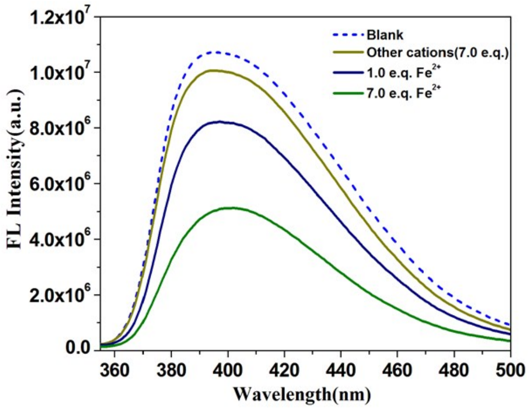

(e)

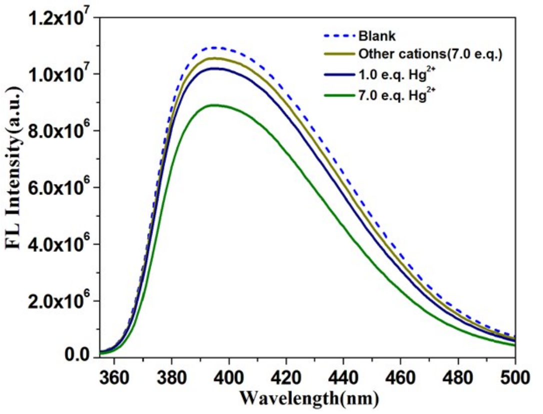

(f)

Fig. S11 (a−f) The relative fluorescence intensity of a 10^{-5} M solution of Cd-P upon addition of 1.0 and 7.0 equiv of Co^{2+} ions, Cu^{2+} ions, Fe^{3+} ions, Ni^{2+} ions, Fe^{2+} ions and Hg^{2+} ions in the presence of 7.0 equiv of background ions (M^{n+}) in 5 mM Tris-HCl/NaCl buffer (pH 7.0) with pH 7.0. λ_{ex}: 318 nm, λ_F: 400 nm, slit width: 4 nm.

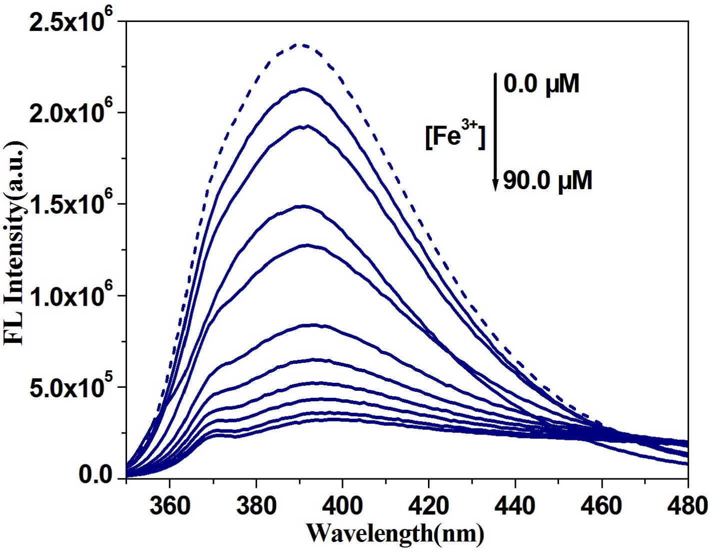

Fig. S12 Luminescence quenching of Tb^{3+}@Cd-P in 5 mM Tris-HCl/NaCl buffer (pH 7.0) with gradual addition of 1 mM solution of Fe^{3+} ions. λ_{ex}: 328 nm, λ_{F}: 390 nm, slit width: 4 nm.

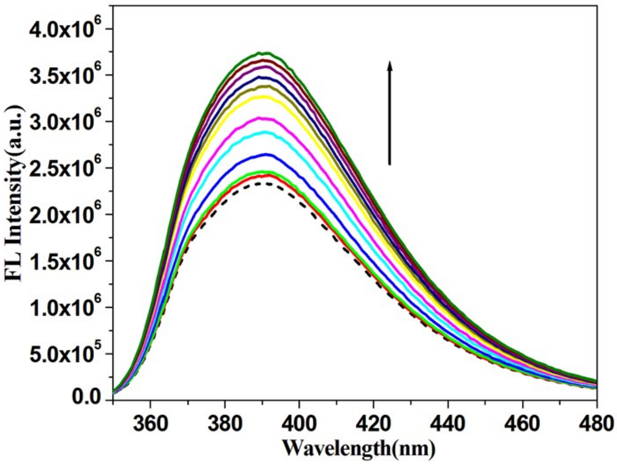

Fig. S13 Luminescence enhancement of Tb^{3+}@Cd-P in 5 mM Tris-HCl/NaCl buffer (pH 7.0) with gradual addition of 1 mM solution of Fe^{2+} ions. λ_{ex}: 328 nm, λ_{F}: 390 nm, slit width: 4 nm.

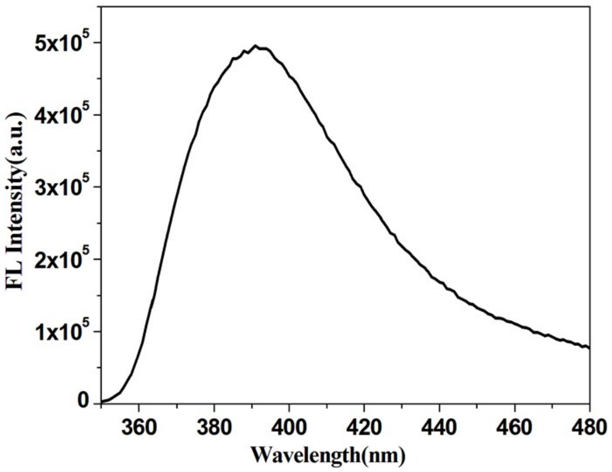

Fig. S14 liquid-state luminescent spectrum of $(NH_4)_2Fe(SO_4)_2$ in H_2O.

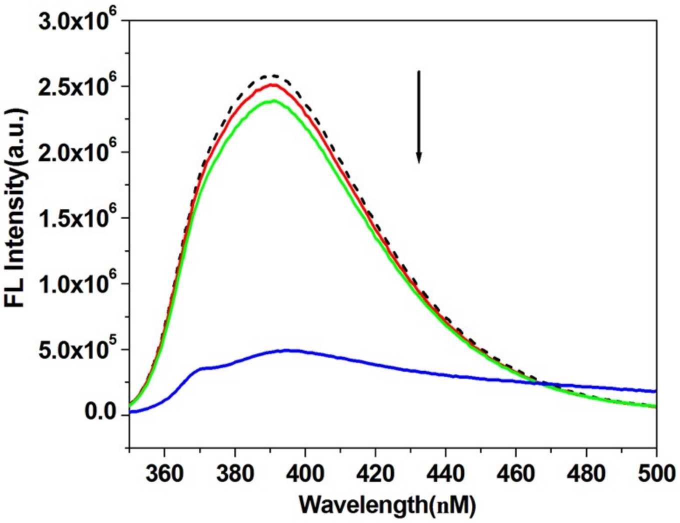

Fig. S15 Comparison of the luminescence intensity of Tb^{3+}@Cd-P in 5 mM Tris-HCl/NaCl buffer (pH 7.0): after addition of mixed ions (Na^+, Ag^+, Cd^{2+}, Zn^{2+}, Pb^{2+}, Ca^{2+}, Cu^{2+}, Co^{2+}, Ni^{2+}, Sn^{2+}, Mg^{2+}, Hg^{2+}, Fe^{2+}, Mn^{2+}; total concentration of mixed metal ions is 70 μM), and followed by addition of Fe^{3+} ions ($[Fe^{3+}]$ = 5 or 70 μM). λ_{ex}: 328 nm, λ_F: 390 nm, slit width: 4 nm.

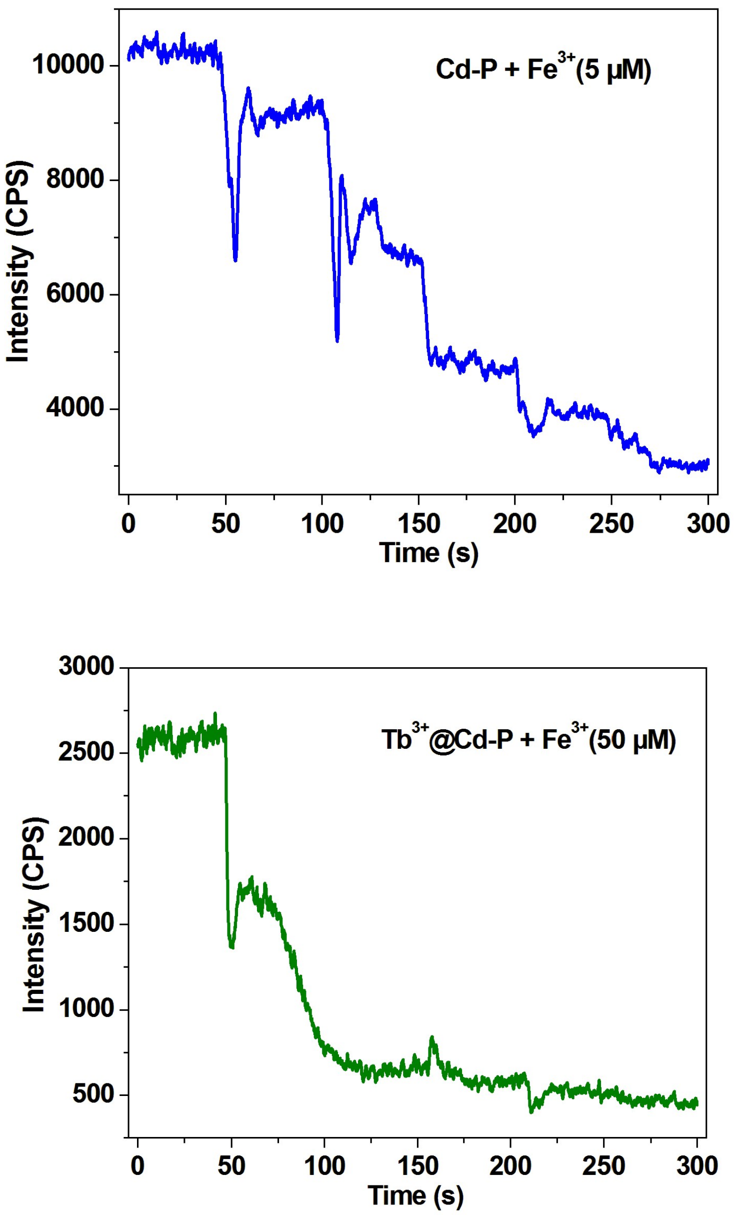

Fig. S16 The variation of luminescent intensity of Cd-P at 400 nm with immersion time in 5 μM $Fe(NO_3)_3$ aqueous solution (blue); Tb^{3+}@Cd-P at 390 nm with immersion time in 50 μM $Fe(NO_3)_3$ aqueous solution (olive).

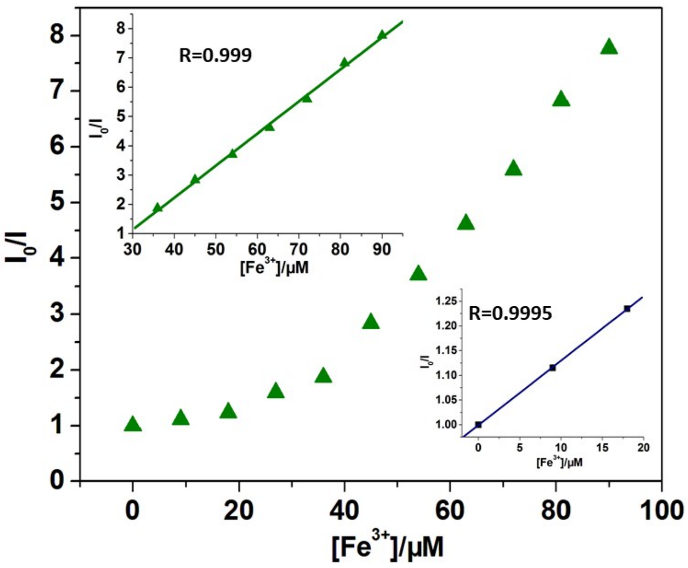

Fig. S17 I_0/I of Tb^{3+}@Cd-P versus Fe^{3+} ions concentration. λ_{ex}: 328 nm, λ_F: 390 nm, slit width: 4 nm.

Table S3. Comparison of the probes with literature reports for sensing Fe^{3+}.

S. No	Probe	LOD (M)	Ref
1	Mg-CP probe	4.7×10^{-4}	S1
2	Zn-L-MOF probe	6.4×10^{-6}	S2
3	Zn(II)-based MOF probe	2×10^{-6}	S3
4	Ln(III)-MOF probe	10^{-6}	S4
5	Europium-Based MOF probe	0.793×10^{-6}	S5
6	{[Eu(Hdcppa)(H_2O)$_2$]·H_2O}n probe	10^{-6}	S6
7	Tb^{3+}@Cd-P probe	6.6×10^{-7}	This work
8	Cd-P probe	4.7×10^{-8}	This work

S1. Wu, Z. F.; Gong, L. K.; Huang, X. Y. A Mg-CP with in Situ Encapsulated Photochromic Guest as Sensitive Fluorescence Sensor for Fe^{3+}/Cr^{3+} Ions and Nitro-Explosives. *Inorg. Chem.* **2017**, *56*, 7397−7403.

S2. Yu, C. Y.; Sun, X. D.; Zou, L. F.; Li, G. H.; Zhang, L. R.; Liu, Y. L. A Pillar-Layered Zn-LMOF with Uncoordinated Carboxylic Acid Sites: High Performance for Luminescence Sensing Fe^{3+} and TNP. *Inorg. Chem.* **2019**, *58*, 4026−4032.

S3. Lv, R.; Li, H.; Su, J.; Fu, X.; Yang, B. Y.; Gu, W.; Liu, X. Zinc Metal−Organic Framework for Selective Detection and Differentiation of Fe(III) and Cr(VI) Ions in Aqueous Solution. *Inorg. Chem.* **2017**, *56*, 12348−12356.

S4. Zhang, Q. S.; Wang, J.; Alexander, M. K.; Dou, W.; Xu, C.; Xu, C. L.; Yang, L. Z.; Fang, R.; Liu, W. S. Multifunctional Ln−MOF Luminescent Probe for Efficient Sensing of Fe^{3+},Ce^{3+}, Acetone. *ACS Appl. Mater. Interfaces.* **2018**, *10*, 23976−23986.

S5. Purna, C. R.; Mandal, S. Europium-Based Metal−Organic Framework as a Dual Luminescence Sensor for the Selective Detection of the Phosphate Anion and Fe^{3+} Ion in Aqueous Media. *Inorg. Chem*. **2018**, *57*, 11855−11858

S6. Zhang, H. J.; Fan, R. Q.; Chen, W.; Fan, J. Z.; Dong, Y. W.; Song, Y.; Du, X.; Wang, P.; Yang, Y. L. 3D Lanthanide Metal−Organic Frameworks Based on Mono-, Tri-, and Heterometallic Tetranuclear Clusters as Highly Selective and Sensitive Luminescent Sensor for Fe^{3+} and Cu^{2+} Ions. *Cryst. Growth Des*. **2016**, *16*, 5429−5440.

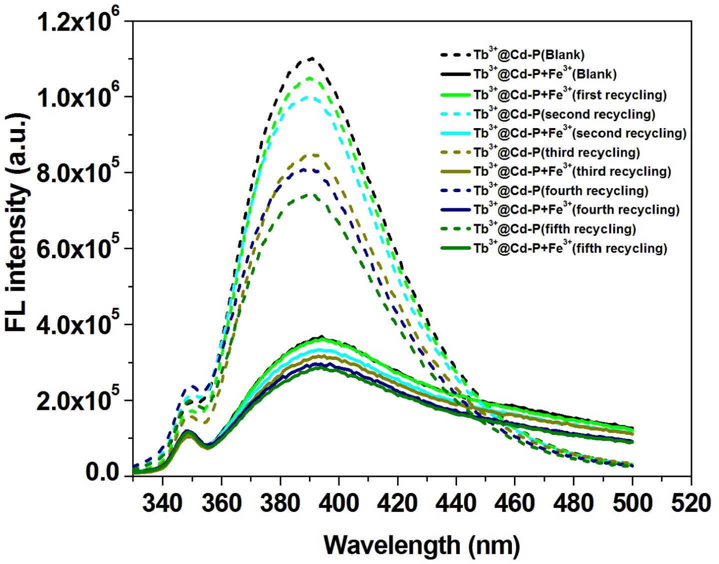

Fig. S18 Five regeneration cycles for detection of Fe^{3+} ions by Tb^{3+}@Cd-P. λ_{ex}: 328 nm, λ_F: 390 nm, slit width: 4 nm.

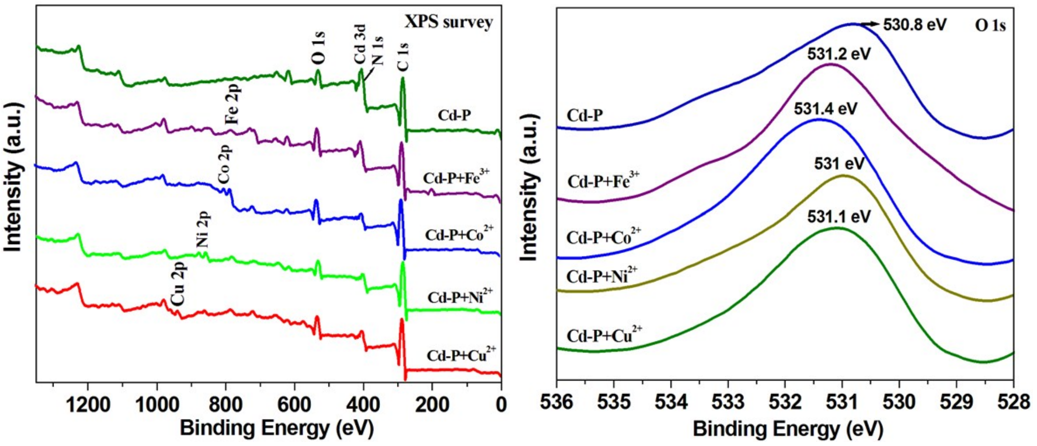

(a) (b)

Fig. S19 XPS spectra of Cd-P, and after added Cu^{2+}, Co^{2+}, Ni^{2+}, Fe^{3+} samples: (a) the survey spectrum, and (b) the high resolution XPS spectra of O 1s.

Table S4 The detailed ICP studies of Cd-P and Tb^{3+}@Cd-P.

Sample	Tb^{3+}(ppm)	Hg^{2+}(ppm)	Sm^{3+}(ppm)	Fe^{3+}(ppm)	Cr^{3+}(ppm)	Eu^{3+}(ppm)	Cu^{2+}(ppm)	Co^{3+}(ppm)
Tb^{3+}@Cd-P	Below detection limit							
Cd-P+Cu^{2+}							0.0656	
Cd-P+Fe^{3+}				0.3564				
Cd-P+Hg^{2+}		0.0077						
Cd-P+Co^{3+}								0.0933
Cd-P+Sm^{3+}			Below detection limit					
Cd-P+Eu^{3+}						Below detection limit		
Cd-P+Cr^{3+}					0.1696			
Tb^{3+}@Cd-P+Fe^{3+}	Below detection limit			0.0489				
detection limit / ppm	0.03		0.05			0.01		

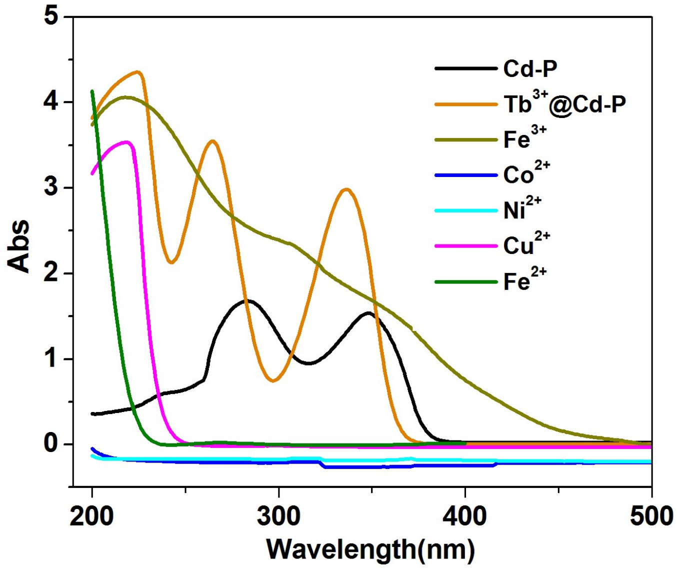

Fig. S20 UV spectra of Cu^{2+}, Fe^{3+}, Fe^{2+}, Co^{2+}, Ni^{2+}, Cd-P and Tb^{3+}@Cd-P in DMF-DMSO (3:1).

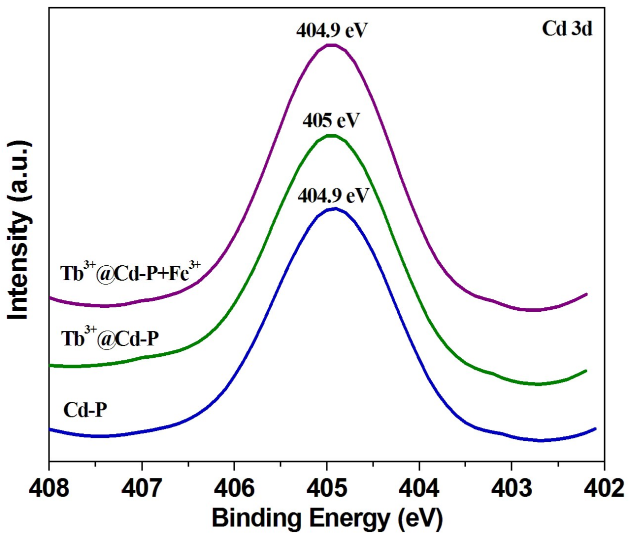

Fig. S21 The high resolution XPS spectra for Cd-P, Tb^{3+}@Cd-P and Tb^{3+}@Cd-P+Fe^{3+} samples of Cd 3d.

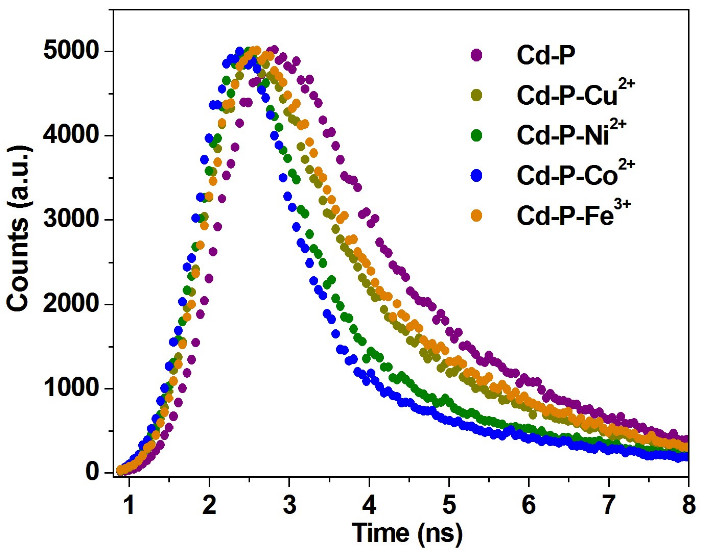

Fig. S22 Temporal fluorescence decay of 100 μM Cd-P with 100 μM Cu^{2+}, Co^{2+}, Ni^{2+} and Fe^{3+} in 5 mM Tris-HCl/NaCl buffer (pH 7.0) excited at 318 nm and monitored at 400 nm; The data are obtained at 54.9 ps per point.

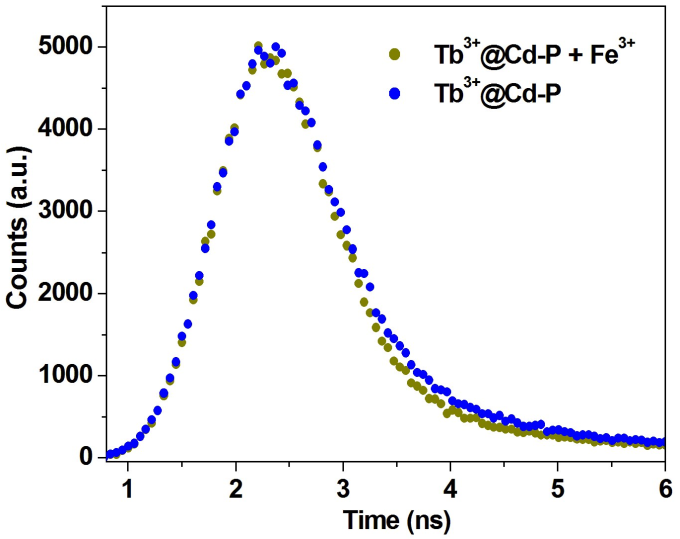

Fig. S23 Temporal fluorescence decay of 100 μM Tb^{3+}@Cd-P with 100 μM Fe^{3+} in 5 mM Tris-HCl/NaCl buffer (pH 7.0) excited at 328 nm and monitored at 390 nm; The data are obtained at 54.9 ps per point.

Table S5 Comparison of lifetimes of Cd-P, Cd-P-Cu^{2+}, Cd-P-Fe^{3+}, Cd-P-Co^{2+}, Cd-P-Ni^{2+}, Tb^{3+}@Cd-P and Tb^{3+}@Cd-P-Fe^{3+}.

CPs	τ_1 (ns)	B_1 (%)	τ_2 (ns)	B_2 (%)	τ (ns)
Cd-P	24	4.58	1.95	95.42	2.960
Cd-P-Cu^{2+}	0.68	31.57	2.59	68.43	1.987
Cd-P-Fe^{3+}	0.93	26.74	3.42	73.26	2.754
Cd-P-Co^{2+}	1.13	34.95	2.38	65.05	1.943
Cd-P-Ni^{2+}	1.12	28.39	2.32	71.61	1.979
Tb^{3+}@Cd-P	0.54	47.45	2.22	52.55	1.423
Tb^{3+}@Cd-P-Fe^{3+}	0.52	56.87	2.34	43.13	1.305
